# HfZrO-based synaptic resistor circuit for a Super-Turing intelligent system

**DOI:** 10.1126/sciadv.adr2082

**Published:** 2025-02-28

**Authors:** Jungmin Lee, Rahul Shenoy, Atharva Deo, Suin Yi, Dawei Gao, David Qiao, Mingjie Xu, Shiva Asapu, Zixuan Rong, Dhruva Nathan, Yong Hei, Dharma Paladugu, Jian-Guo Zheng, J. Joshua Yang, R. Stanley Williams, Qing Wu, Yong Chen

**Affiliations:** ^1^ Departments of Materials Science and Engineering, Mechanical and Aerospace Engineering, Electrical and Computer Engineering, California NanoSystems Institute, University of California, Los Angeles, Los Angeles, CA 90095, USA.; ^2^ Department of Electrical and Computer Engineering, Texas A&M University, College Station, TX 77843, USA.; ^3^ Irvine Materials Research Institute, University of California, Irvine, Irvine, CA 92697, USA.; ^4^ Department of Electrical and Computer Engineering, University of Massachusetts, Amherst, MA 01003, USA.; ^5^ Department of Electrical and Computer Engineering, University of Southern California, Los Angeles, CA 90089, USA.; ^6^ Air Force Research Lab, Information Directorate, Rome, NY 13441, USA.

## Abstract

Computers based on the Turing model execute artificial intelligence (AI) algorithms that are either programmed by humans or derived from machine learning. These AI algorithms cannot be modified during the operation process according to environmental changes, resulting in significantly poorer adaptability to new environments, longer learning latency, and higher power consumption compared to the human brain. In contrast, neurobiological circuits can function while simultaneously adapting to changing conditions. Here, we present a brain-inspired Super-Turing AI model based on a synaptic resistor circuit, capable of concurrent real-time inference and learning. Without any prior learning, a circuit of synaptic resistors integrating ferroelectric HfZrO materials was demonstrated to navigate a drone toward a target position while avoiding obstacles in a simulated environment, exhibiting significantly superior learning speed, performance, power consumption, and adaptability compared to computer-based artificial neural networks. Synaptic resistor circuits enable efficient and adaptive Super-Turing AI systems in uncertain and dynamic real-world environments.

## Teaser

Synaptic resistor circuits for Super-Turing AI systems feature low power consumption, fast learning, and agile adaptability.

## INTRODUCTION

The development of artificial intelligence (AI) systems has predominantly relied on computers. Following the Turing model (*1*), computers can accurately execute predetermined inference algorithms programmed by humans and/or derived from machine learning processes (*2*–*11*). Present neuromorphic computing circuits, using digital electronics (*4*–*7*) or analog resistive devices such as floating-gate transistors (*12*–*14*), memristors (*15*–*19*), ferroelectric junctions (*20*) and diodes (*21*), and phase-change memory resistors (*22*), can execute inference algorithms previously programmed through learning (training). All computing circuits can only execute algorithms that are explicitly predefined, and these algorithms remain fixed and cannot be modified during the computing processes in response to environmental changes. AI systems need to improve their ability to handle diverse and complex conditions by expanding their prelearning domains through the use of “big data,” which significantly increases the time and energy required for learning (*2*–*11*). Consequently, the computationally intensive learning processes are often carried out on large-scale, power-hungry off-site computers to derive optimal inference algorithms, which are then executed on edge computers with a stringent power budget. With their accurate execution of the predefined inference algorithms, AI systems, such as self-driving cars (*8*), drones (*9*, *10*), robotic systems (*23*), and large language models (*11*), may outperform humans within their learning domains. However, these AI systems lack the adaptability of the human brain and are prone to failure in unknown environments that extend beyond their learning domains (*8*–*11*, *23*). For example, accidents occurred when self-driving cars operated in environments beyond their prelearned domains (*8*). Navigating a self-driving drone in unpredictable, complex environments, such as those with obstacles and strong winds, incurs complex turbulence, instability, and collisions, making it one of the most significant challenges in the development of self-driving aerial vehicles (*10*). Attempting to expand learning domains through the utilization of even more data through the utilization of “big data” from complex changing environments is expensive, inefficient, and unsustainable (*24*, *25*), and the inference algorithms derived from finite learning domains restrict their performance in real-world environments with infinite variations.

The brain has long inspired the development of AI technology. Neurobiological networks in the brain process massive spike signals input to presynaptic neurons, generating currents through synapses and triggering output spikes at postsynaptic neurons in analog parallel mode (*26*–*30*). Human brains can perform algorithmic computations in Turing mode, following prelearned algorithms that remain fixed during the computations (*31*–*33*). However, the brain can also process information without predefined algorithms by learning in real time; its synaptic connections can be modified while performing a task (*26*, *28*, *30*), referred to as Super-Turing computing (*31*–*33*). In neurobiological circuits, synaptic weights (conductance) can be modified during concurrent inference and learning processes by following a learning rule known as spike timing–dependent plasticity (STDP) (Materials and Methods) (*28*, *30*). The ability to simultaneously infer and learn sets the brain apart from computers, leading to significantly shorter learning latency and greater adaptability to dynamically changing environments compared to computers (*34*). The brain often computes based on previously learned experiences but switches to a Super-Turing mode when encountering unexpected conditions that require new learning and adaptation (*32*). For instance, self-driving cars can operate within their prelearned domains, but when they encounter new conditions, human drivers must intervene, devising solutions using their concurrent inference and learning abilities. Although the Super-Turing computing models have been postulated theoretically (*31*, *32*), they did not address the concurrent inference and learning functionality of neurobiological circuits. Experimentally, neuromorphic computing circuits based on either digital transistors (*4*–*7*) or analog resistive devices (*12*–*22*) have been typically operated in the Turing computing mode with sequential learning and inference processes. Various learning algorithms, such as STDP, have been successfully implemented in the circuits of analog resistive devices by applying specific “learning” signals to modify the device conductance; the inference algorithm must be executed sequentially, rather than concurrently, by applying different signals to prevent changes in the device conductance (*12*, *13*, *15*–*19*, *22*). For instance, the memristor conductance was modified according to the STDP learning rule by applying voltage pulses with amplitudes of ±1.7 V, whereas the conductance was read out by applying voltage pulses with an amplitude of 0.4 V to prevent changes in the device conductance (*16*). In previously reported two-terminal ferroelectric tunnel junctions (*20*) and ferroelectric diodes (*21*), device conductance could be tuned for learning with high-magnitude voltages, whereas low-magnitude voltages were used for reading or inference to avoid altering conductance. They cannot perform learning and inference simultaneously. The existing neuromorphic circuits still operated in the Turing mode, and the inference algorithm executed in these circuits cannot be adjusted for learning during inference. The key for Super-Turing computing is to be able to adjust weights in real time during inference. How can we emulate concurrent learning and inference in an electronic system?

In this study, we initially introduce a model of an intelligent system based on a synaptic resistor (synstor) circuit (*35*–*37*) with the concurrent inference and learning functionality, operating in parallel analog Super-Turing mode. We fabricated a circuit of Hf_0.5_Zr_0.5_Ο_2_-based synstors to implement the concurrent inference and learning. We conducted experiments to navigate an aerial drone toward a target position by avoiding obstacles using the synstor circuit and human operators and a computer-based artificial neural network (ANN), in a simulated aerodynamic environment with time-varying strong winds. The experimental results demonstrated that the synstor circuit and human operators significantly outperformed the ANN in terms of learning speed, performance, power consumption, and adaptability to the changing environment.

## RESULTS

### Super-Turing intelligent system based on a synstor circuit

**Fig. 1.**
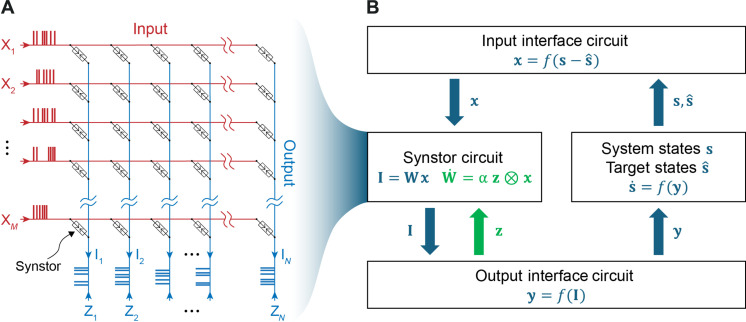
Synstor circuit for intelligent systems. (**A**) Schematic diagram showing a crossbar synstor circuit composed of *M* × *N* synstors connected with *M* input electrodes and *N* output electrodes. **x** denotes a vector with element $x_{m}$ as the voltage pulses applied to the *m*th input electrode, **z** denotes a vector with element $z_{n}$ as the voltage pulses applied to the *n*th output electrode, and **I** denotes a vector with element $I_{n}$ as the current induced at the *n*th output electrode. (**B**) Sensors detect deviations of the states of a system (**s**) from targeted states ($\hat{s}$), denoted as $s-\hat{s}$, and convert them to input voltage pulses (**x**) using an interface circuit. The output currents, I=Wx, triggered by **x** from the synstor circuit flow into an output interface circuit, generating output voltage pulses (**y**) applied on actuators to modify **s**, as well as voltage pulses (**z**) applied on the output electrodes of the synstor circuit to modify the synstor conductance matrix (**W**) according to the learning rule $\overset{\cdot }{W}=\alpha \;z⊗x$.

Similar to a neurobiological network connected by synapses, a circuit composed of *M* input and *N* output electrodes connected by *M* × *N* synstors is depicted in Fig. 1A. Voltage pulses (**x**) applied at the input electrodes induce currents (**I**) through the synstors at the output electrodes to execute an inference function

I=W(t) x (1)

where W(t) denotes the conductance matrix of the synstors. The excitatory (or inhibitory) currents (**I**) trigger (or inhibit) the voltage pulses (**y**) applied on actuators via neuron and interface circuits to modify the states of a system (**s**). The deviations of **s** from targeted states $\hat{s}$, denoted as $s-\hat{s}$, are detected by sensors and converted to **x** by interface circuits (Fig. 1B). The aim is to minimize the objective function $F=\frac{1}{2}{\left(s-\hat{s}\right)}^{2}$, tuning **s** toward $\hat{s}$.

Unlike standard ANNs, where the weights remain unchanged during the execution of their inference functions, the W(t) in the synstor circuit, similar to a neurobiological network, may be modified during the inference process by following a correlative learning rule (*26*,*28*, *30*)

$$\frac{dW}{\mathrm{dt}}=\alpha \;z\left(t\right)⊗x\left(t\right)$$ (2)

where α denotes a learning coefficient, **z** denotes voltage pulses triggered at the output electrodes of the circuit, and z(t)⊗x(t) represents the outer product between z(t) and x(t). The STDP learning rule (*28*, *30*) can also be formulated as Eq. 2, where the temporal mean $\bar{z}=0$ (Materials and Methods and Eq. 3) and the temporal covariance between $z_{n}$ and $y_{n}$, $\bar{z_{n}y_{n}}\leq 0$ (Materials and Methods and Eq. 4). When z in Eq. 2 satisfies the conditions $\bar{z}=0$ and $\bar{z_{n}y_{n}}\leq 0$, the concurrent execution of the inference (Eq. 1) and learning (Eq. 2) in the synstor circuit results in the decrease in the objective function F [i.e., $\bar{\left(\frac{\mathrm{dF}}{\mathrm{dt}}\right)}\leq 0$; Materials and Methods and Eq. 5]. When $\bar{\left(\frac{\mathrm{dF}}{\mathrm{dt}}\right)}<0$, W deviates from $\hat{W}$ due to environmental, system, or circuit changes, and W is adjusted toward $\hat{W}$ to decrease $\bar{F}$ during concurrent learning and inference processes. In this case, the circuit operates in Super-Turing mode. When the covariance between z and x, $\bar{z⊗x}=0$, then $\bar{\frac{dW}{\mathrm{dt}}}=0$, $\bar{\left(\frac{\mathrm{dF}}{\mathrm{dt}}\right)}=0$, and $W=\hat{W}=\text{arg}\;\underset{W}{\text{min}}F$ remain unchanged during the execution of the inference function, $I=\hat{W}x$, and the circuit operates in the Turing mode. To execute the correlative learning rule ($\overset{\cdot }{W}=\alpha \;z⊗x$) by applying various z pulses, different learning functions in all three major machine learning paradigms (unsupervised, supervised, and reinforcement learning) can be implemented in multilayer synstor circuits (*29*).

### Synstor circuit based on HfZrO ferroelectric materials

**Fig. 2.**
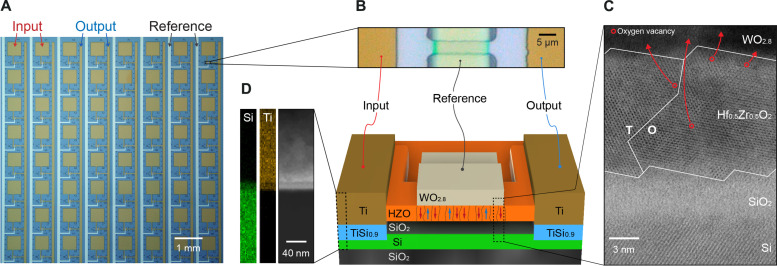
Structure of a synstor circuit. (**A**) A top-view optical image shows an 8 × 8 synstor crossbar composed of eight rows of Ti input electrodes, eight columns of Ti output electrodes, and eight columns of WO_2.8_ reference electrodes. (**B**) Top-view optical image (top) and a three-dimensional (3D) schematic illustration (bottom) of a synstor composed of a Si channel surrounded by SiO_2_ layers, a WO_2.8_ reference electrode grown on a Hf_0.5_Zr_0.5_O_2_ layer on the SiO_2_ layer on the Si channel. The Si channel is connected with a TiSi_0.9_ layer and Ti input and output electrodes. The Hf_0.5_Zr_0.5_O_2_ layer is composed of multiple ferroelectric domains, indicated by red or blue arrows, and dielectric domains, depicted without arrows. (**C**) Cross-sectional STEM image across the WO_2.8_/Hf_0.5_Zr_0.5_O_2_/SiO_2_/Si heterojunction with tetragonal (T) and orthorhombic (O) ferroelectric phases in the Hf_0.5_Zr_0.5_O_2_ layer. The arrows illustrate the migrations of oxygen vacancies, represented as red open circles, from the Hf_0.5_Zr_0.5_O_2_ layer to the WO_2.8_ layer. (**D**) A cross-sectional STEM image (right) and EDX chemical maps of Ti and Si elements (left) across the Ti/TiSi_0.9_/Si heterojunction are displayed.

The synstor circuit poses a challenging demand for materials and devices that can facilitate the precise, reliable, fast, and repetitive execution of both analog inference and learning concurrently. In this work, we fabricated synstors by integrating a WΟ_2.8_/Hf_0.5_Zr_0.5_Ο_2_ heterojunction with a Si channel in each device. Hf oxides have emerged as promising ferroelectric materials, enabling scalable, Si-compatible nonvolatile analog memory and neuromorphic devices (*38*–*42*). As shown in Fig. 2A, a crossbar synstor circuit was fabricated by following the process described in Materials and Methods and illustrated in fig. S1. The structure and materials of the synstors were characterized by scanning transmission electron microscopy (STEM), energy-dispersive x-ray (EDX) spectroscopy, and electron energy loss spectroscopy (EELS) and are shown in Fig. 2 (B to D), and fig. S2. A 40-μm-long 220-nm-thick Si channel with a boron doping concentration of 10^15^/cm^3^ was made from a single-crystal Si layer on a 3-μm-thick SiO_2_ layer. A 14-μm-long 80-nm-thick WO_2.8_ reference electrode was fabricated on a 12.6-nm-thick Hf_0.5_Zr_0.5_O_2_ layer on a 3.5-nm-thick SiO_2_ dielectric layer on the Si channel. The Hf_0.5_Zr_0.5_O_2_ layer was composed of ferroelectric orthorhombic, dielectric tetragonal and monoclinic phases (*40*). A 12-nm-thick metallic TiSi_0.9_ layer was sandwiched between the Ti input/output electrodes and Si channel and formed a Schottky contact to the Si channel (*43*).

**Fig. 3.**
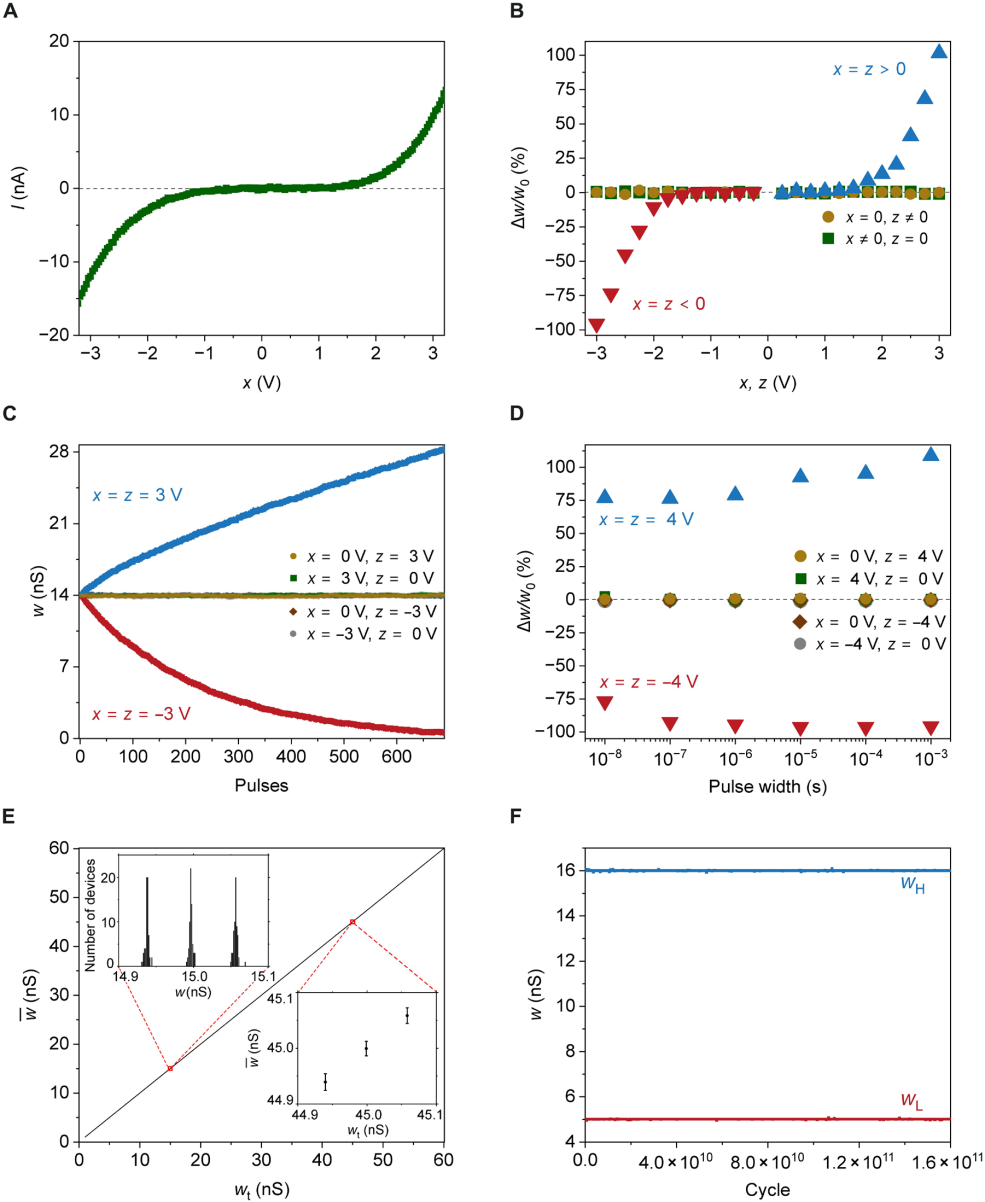
Electric properties of synstors. (**A**) The current, I, on the output electrode of a synstor is displayed versus voltage, x, applied on the input electrode of the synstor with respect to its grounded output and reference electrodes. (**B**) The percentage changes in the synstor conductance, (∆*w*/*w*_0_) × 100%, are plotted versus various x and z pulses concurrently applied on the input and output electrodes. (**C**) The changes in the synstor conductance, w, induced by various x and z pulses are plotted against the numbers of applied pulses. (**D**) The percentage changes in conductance w, (∆*w*/*w*_0_) × 100%, of a synstor are shown against the widths of the applied x and z pulses on its input and output electrodes, respectively, after the synstor experienced pulses with a total duration of 500 ms and amplitudes x=z=−4 V (red triangles), x=z=4 V (blue triangles), x=0 and z=4 V (brown circles), x=4 V and z=0 (green squares), x=−4 V and z=0 (gray circles), and x=0 and z=−4 V (brown diamonds). (**E**) The average analog conductance values, $\bar{w}$, of 64 synstors are plotted against 1000 targeted analog conductance values, $w_{t}$, evenly separated by $\Delta w_{t}=59\;\text{pS}$. The inset in the top-left corner shows the distribution of the synstor conductance values when $w_{t}=14.938$, 14.997, and 15.056 nS. The inset in the bottom-right corner shows $\bar{w}$ with $\pm 3\sigma_{w}$ SDs against $w_{t}$ at 44.940, 44.999, and 45.058 nS. (**F**) A synstor was iteratively modified to its high (*w*_*H*_ = 16 nS) and low ($w_{L}=5\;\text{nS}$) conductance values, which are displayed as blue and red squares, respectively, against the modification cycles.

To emulate synapses grounded to the cerebrospinal fluid in neurobiological circuits, the reference electrodes of synstors were always grounded during the tests. When voltage pulses with an amplitude *x* were applied on a synstor in the circuit to induce current *I* through the synstor, a nonlinear *I* − *x* relation (Fig. 3A) revealed the Schottky barriers between the Si channel and TiSi_0.9_ layers (*43*). The synstor circuit executes the inference function I=Wx (Eq. 1), when multiple voltage pulses, **x**, are applied on the input electrodes. The learning functionality of synstors was tested by the application of *x* and *z* pulses with various amplitudes, numbers, and widths on their input and output electrodes, respectively. After a synstor experienced 690 paired *x* and *z* pulses with amplitudes *x* = *z* and a width of 2 ms, the percentage changes in *w*, (∆*w*/*w*_0_) × 100%, increased from 0 to 102% when the voltage amplitudes increased from 1.2 to 3 V; $∆w/w_{0}$ decreased from 0 to −95% when *x* and *z* decreased from −1.2 to −3 V; when the voltage amplitudes −1.2 V ≤ *x* = *z* ≤ 1.2 V, $|∆w/w_{0}|\approx 0$ (Fig. 3B). The synstor conductance was gradually tuned in analog mode by applying individual paired pulses with amplitudes x=z=±3 V and a width of 2 ms (Fig. 3C). After the synstor experienced x or z pulses with −3 V≤x≤3 V and z=0 V, or x=0 V and −3 V≤z≤3 V, $|∆w/w_{0}|\approx 0$ (Fig. 3, B and C). Synstors in the circuit executed the learning function, ${\overset{\cdot }{w}}_{\mathrm{nm}}=\alpha \;z_{n}\;x_{m}$ (Eq. 2) with the modification coefficient α≥0 when $3\;V\geq x_{m}=z_{n}\geq 1.2\;V$ and α≤0 when $-3\;V\leq x_{m}=z_{n}\leq -1.2\;V$. When $z_{n}=0$, and $3\;V\geq x_{m}\geq 1.2\;V$, or $-3\;V\leq x_{m}\leq -1.2\;V$, the circuit executed the learning function ${\overset{\cdot }{w}}_{\mathrm{nm}}=z_{n}\;x_{m}=0$, and the inference function $I_{\mathrm{nm}}=w_{\mathrm{nm}}\;x_{m}$ concurrently. The operational voltages of synstors fall within the operational voltage range (±3.8 V) of 22-nm CMOS (complementary metal-oxide semiconductor) circuits with HfZrO-based ferroelectric transistors (*40*).

The synstor conductance was also effectively modified by applying paired pulses with amplitudes x=z=±4 V and widths ranging from 10 ns to 1 ms (Fig. 3D), indicating that the device conductance can be modified within 10 ns, which has also been observed in HfZrO-based transistors (*40*). The analog learning accuracy was assessed through the fine-tuning of 64 synstors in the circuit precisely toward 1000 distinct values, $w_{t}$, ranged between 1 and 60 nS and separated evenly by $\Delta w_{t}=59\;\text{pS}$ by applying a train of paired $x_{m}=z_{n}$ pulses (Materials and Methods). As shown in Fig. 3E and fig. S3 (A and B), the disparities between the average conductance values of the 64 synstors and their respective $w_{t}$, $|\bar{w}-w_{t}|$, are smaller than 1.5 pS or 2.5% of $\Delta w_{t}$, and the SDs $\sigma_{w}$ at different $\bar{w}$ are below 6 pS or 10% of $\Delta w_{t}$. As shown in fig. S3C, the 64 as-fabricated devices initially exhibited considerable variation in conductance, with an average conductance of 2.7 nS and an SD of 2.1 nS, but this variation was substantially reduced to an average of 1.531 nS and an SD of 0.001 nS after tuning the devices to a target conductance value ($w_{t}=1.5315\;\text{nS}$). The uniformity of conductance values w for the 64 synstors, tuned to the targeted conductance value ($w_{t}=1.5315\;\text{nS}$, $\bar{w}=1.531\;\text{nS}$, and $\sigma_{w}=0.001\;\text{nS}$), is significantly better than that of carbon nanotube (CNT)–based ($\bar{w}=1.90\;\text{nS}$ and $\sigma_{w}=0.45\;\text{nS}$) (*35*) and Al oxide-based ($\bar{w}=2.219\;\text{nS}$ and $\sigma_{w}=0.062\;\text{nS}$) (*37*) synstors (fig. S3D). The 1000 dynamically tunable conductance levels and the precise conductance modification accuracy of 36 pS ($6\sigma_{w}$) could be attributed to the progressive switching of the multiple individual nanoscale ferroelectric domains in the Hf_0.5_Zr_0.5_O_2_ layer (Fig. 2C) (*39*, *41*). To assess their learning endurance, synstors were iteratively adjusted to their high ($w_{t}=16\;\text{nS}$) and low ($w_{t}=5\;\text{nS}$) conductance values (Materials and Methods). No degradation in device conductance was observed over 1.6 × 10^11^ switching cycles (Fig. 3F). Synstors were iteratively tuned to their maximum (60 nS) and minimum (0.6 nS) conductance values. No degradation in device conductance was observed over 1.1 × 10 switching cycles (fig. S3E). The distributions of the conductance values across the different tuning cycles are shown in fig. S3F, with $\bar{w}=59.9998\;\text{nS}$ and $\sigma_{w}=0.0907\;\text{nS}$ for the maximum conductance ($w_{t}=60\;\text{nS}$) and $\bar{w}=0.6314\;\text{nS}$ and $\sigma_{w}=0.0628\;\text{nS}$ for the minimum conductance ($w_{t}=0.6\;\text{nS}$). HZO-based devices typically degraded after ∼10^6^ switching cycles, mainly induced by oxygen vacancies in the Hf_0.5_Zr_0.5_O_2_ layer (*39*, *40*). In the WO_2.8_/Hf_0.5_Zr_0.5_O_2_ heterojunction of the synstor, the oxygen vacancies with higher defect energy levels in the Hf_0.5_Zr_0.5_O_2_ layer tended to migrate toward the W oxide layer with lower defect energy levels (Fig. 2C) (*44*), significantly enhancing the switching cycles of the Hf_0.5_Zr_0.5_O_2_ layer and converting the W oxide layer to metallic reference electrode (*45*). The current (I) through the synstor was measured as a function of input voltage (x) over a temperature range of 24° to 80°C (Materials and Methods). No significant hysteresis was observed in the I−x curves, indicating that the input voltage alone does not change the device conductance (fig. S3G). The conductance increases with increasing temperature due to the higher carrier concentration in the Si semiconducting channel. However, the conductance can be adjusted to maintain a constant target value across 24° to 80°C by applying paired x and z pulses, compensating for temperature-induced changes in carrier concentration (fig. S3H). The current-voltage characteristics and resistance of a WO_2.8_ resistor were measured across a temperature range of 80 to 320 K (Materials and Methods). No notable change in resistance with temperature was observed in the Arrhenius plot (fig. S4A), and the linear current-voltage curves (fig. S4B) suggests the metallic conduction mechanism in WO_2.8_ film. The resistivity of the WO_2.8_ layer is estimated to be 5.0 × 10^−6^ ohms · m, which is comparable with previously reported values for defected tungsten oxide (*45*) and metals with high resistivity. The conductance values of synstors were measured at 40° and 85°C versus time after the synstors were modified by paired pulses (Materials and Methods and fig. S5, A and B). Similar to the dynamic changes of synaptic weights in neurobiological networks (*28*, *30*), the conductance values of the synstors changed at the initial testing stage, possibly due to incomplete charge compensation in the Hf_0.5_Zr_0.5_O_2_ layer. However, the ferroelectric dipoles and device conductance values remained stable for long-term memory (*46*). The devices tested at 40°C exhibit distinguishable conductance states, whereas those tested at 85°C do not. The higher temperature likely induces significant fluctuations in the electric dipoles within the Hf_0.5_Zr_0.5_O_2_ layer, leading to more random variations in conductance across different devices. However, dynamic adjustment of the device conductance and learning can compensate for and correct these temperature-induced conductance variations, as illustrated in fig. S3H.

**Fig. 4.**
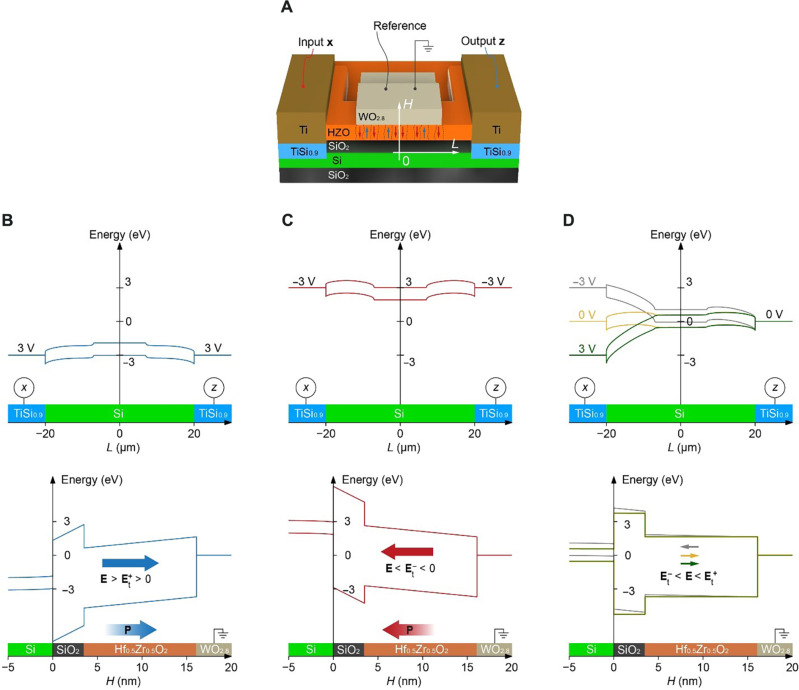
Energy-band structures of a synstor. (**A**) 3D schematic illustration of a synstor composed of a grounded WO_2.8_ reference electrode, Hf_0.5_Zr_0.5_O_2_ layer, SiO_2_ dielectric layer, and Si channel along the *H* axis and a TiSi_0.9_ input electrode, Si channel, and TiSi_0.9_ output electrode along the *L* axis. The Hf_0.5_Zr_0.5_O_2_ layer is composed of multiple ferroelectric domains, indicated by red or blue arrows, and dielectric domains, depicted without arrows. The simulated energy-band structures along the *L* axis (top) and along the *H* axis (bottom) of a synstor under the conditions of (**B**) the potentials at the input electrode x=3 V and the output electrode z=3 V, (**C**) x=z=−3 V, and (**D**) x=3 V and z=0 (green lines), x=−3 V and z=0 (gray lines), and x=z=0 (yellow lines). The top scale (*L*) represents the lateral distance from the input electrode to the output electrode, and the bottom scale (*H*) represents the vertical distance from the Si channel to the W reference electrode (also shown in Fig. 1D). When the electric field, E, in the Hf_0.5_Zr_0.5_O_2_ layer satisfies $E>E_{t}^{+}>0$ in (B), or $E<E_{t}^{-}<0$ in (C), with $E_{t}^{+}$ and $E_{t}^{-}$ as the positive and negative electric threshold fields to switch the polarization of the individual ferroelectric domains in the Hf_0.5_Zr_0.5_O_2_ layer, leading to the changes of the dipole density, ∆P>0 or ∆P<0. When $E_{t}^{-}<E<E_{t}^{+}$ in (D), ∆P≈0.

The polarization (*P*) of a 10-nm-thick Hf_0.5_Zr_0.5_O_2_ film was measured as a function of the applied voltage (*V*) across the film (Materials and Methods). The resulting *P*-*V* curve (fig. S6) demonstrates the ferroelectric properties of the Hf_0.5_Zr_0.5_O_2_ film. The ability of the device to dynamically adjust its conductance in analog mode is attributed to the gradual switching of multiple nanoscale ferroelectric domains within the ferroelectric Hf_0.5_Zr_0.5_O_2_ layer (*39*, *41*). As shown in the simulated band structures of the synstor (Fig. 4), when a pair of positive or negative voltage pulses with magnitudes of *x* = *z* = 3.0 or −3.0 V are applied, an electric field is generated within the Hf_0.5_Zr_0.5_O_2_ layer, reaching approximately **E** ≈ 790 or −790 kV/cm (Fig. 4, A and B). This electric field exceeds the critical positive and negative threshold values, $E>E_{t}^{+}$ or $E<E_{t}^{-}$, required to switch individual nanoscale ferroelectric domains in the Hf_0.5_Zr_0.5_O_2_ layer. Under these conditions, the paired voltage pulses progressively switch the individual ferroelectric domains, altering their ferroelectric dipoles (with ∆P>0 or ∆P<0), and subsequently either attract or repel holes in the p-type Si channel. This adjustment in the ferroelectric dipoles results in an increase or decrease in the synstor conductance in analog mode (Fig. 3C). In contrast, when the synstor is subjected to a single voltage pulse with *x* = 3 V and *z* = 0, or *x* = −3 V and *z* = 0, or in the absence of pulses (*x* = *z* = 0), the electric potential primarily drops across the Schottky junction formed between the Si channel and the TiSi_0.9_ layer (*43*). In this scenario, the electric field within the Hf_0.5_Zr_0.5_O_2_ layer, which is located beyond the Schottky junction, becomes **E** = −4, −153, or −3 kV/cm, respectively (Fig. 4D). Under these conditions, the electric field does not exceed the threshold values ($E_{t}^{-}<E<E_{t}^{+}$), so it cannot modify the ferroelectric dipoles (∆P≈0), and thus, the synstor conductance remains unchanged (Fig. 3, B and C).

### Drone navigated by a synstor circuit and human operators

**Fig. 5.**
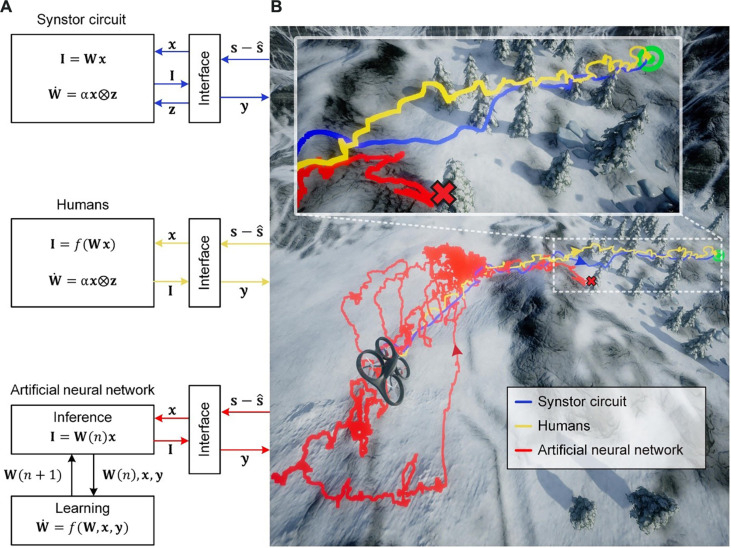
Experiments to negative a drone by a synstor circuit, human operators, and an ANN. (**A**) A synstor circuit (top), humans (middle), and a computer-based ANN (bottom) (**B**) drive a drone toward a target position by avoiding obstacles in a simulated windy environment. The starting point of the flights is marked by a drone image, and the final target position is marked by a green target symbol. The flying traces are shown for the drone driven by the synstor circuit (blue line), the human (yellow line), and the ANN (red line). The inset shows the details in the area near the target with tree obstacles. The red crash symbols indicate that the drone crashed into trees.

A synstor circuit navigated a drone toward a target position by avoiding obstacles in a simulated environment with time-varying strong wind (Materials and Methods, Fig. 5, fig. S7A, and movie S1). Obstacles were detected by a simulated camera on the drone, and a moving target position ($\hat{s}$) was identified to avoid the obstacles and minimize an objective function $F=\frac{1}{2}{\left(s-\hat{s}\right)}^{2}$, with $s-\hat{s}$ as the deviations of the drone position (s) from its target positions (Materials and Methods and fig. S7D). In the windy environment, the speeds and directions of winds changed randomly during the flight (fig. S8A). During the synstor experiments, $s-\hat{s}$ was converted to input voltage pulses, x, by an interface circuit to generate currents via the synstor circuit according to its inference function, I=Wx (Eq. 1). The currents triggered actuation signals, y, via a neuron and an interface circuit to drive the drone. The conductance matrixes (W) of the synstor circuit were initialized to random values before each experiments started (Materials and Methods and fig. S10) to ensure that the circuit had no prior learning experience or predefined function. Concurrently, voltage pulses, z, meeting the conditions $\bar{z}=0$ (Materials and Methods and Eq. 3) and $\bar{z_{n}y_{n}}\leq 0$ (Materials and Methods and Eq. 4) were triggered at the output electrodes of the synstor circuit (Materials and Methods) to modify W according to the learning rule $\overset{\cdot }{W}=\alpha \;z⊗x$ (Eq. 2) during the real-time learning process in a Super-Turing computing mode.

Human operators also navigated a drone toward the target position by avoiding obstacles in the same simulated windy environment (Materials and Methods, Fig. 5, figs. S7B and S8B, and movie S1). The human operators had no prior experience with the drone control system, ensuring that they learned while navigating the drone. During the human experiments, the operators visually saw the analog numbers $s-\hat{s}$ displayed on a monitor and manually triggered an actuation signal y from a keyboard to drive the drone. Concurrently, the synaptic weight matrixes W could be modified using the synaptic learning function (*26*, *28*, *30*) $\overset{\cdot }{W}=\alpha \;z⊗x$ during the real-time learning process.

### Drone navigated by a computer-based ANN

In the control experiments for computer-based ANN to navigate a drone toward the target position by avoiding obstacles in the simulated windy environment, various ANN structures and learning parameters were tested, and the optimal ANN with the shortest learning time was then deployed to navigate the drone (Materials and Methods, Fig. 5, figs. S7C, S8C, and S10, and movie S1). The time required to execute the learning algorithm was significantly longer than that to execute the inference algorithm (fig. S10F); thus, it was infeasible to execute real-time learning on the computer, and the inference and learning had to be executed sequentially. The $s-\hat{s}$ signals were converted to input signals, x, and induced output currents I=W(n) x, triggering actuation signals, y, from the ANN to drive the drone in its *n*th flight episode. The saved experimental data x, y, and W(n) from the *n*th episode were sent back to the computer to execute a reinforcement learning algorithm (*47*) (Materials and Methods) to modify W(n) to W(n+1), which reset the ANN to drive the drone at the (*n* + 1)th flight episode iteratively until the drone crashed into an obstacle or reached the final target (Fig. 5 and fig. S7C).

### Learning time, performance, adaptability, and power consumption

**Fig. 6.**
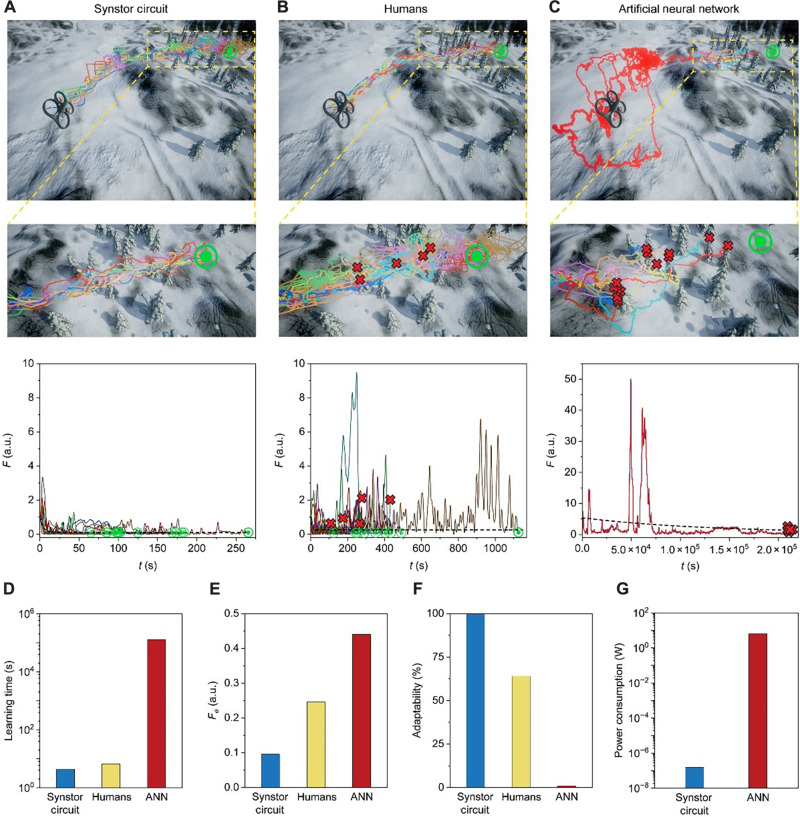
Experimental results of drone navigations. (Top row) The flying traces are shown for a drone driven by (**A**) a synstor circuit, (**B**) human operators, and (**C**) an ANN toward a target position by avoiding obstacles in a simulated windy environment. The starting point of the flights is marked by drone images. (Middle row) The zoom-in images show the details of the flying traces in the area near the target with tree obstacles. (Bottom row) Objective functions, $F=\frac{1}{2}{\left(s-\hat{s}\right)}^{2}$, with $s-\hat{s}$ as the deviations of the drone position (s) from its target position ($\hat{s}$) versus accumulative flight and learning time t. The F−t curves are best fitted by $F\left(t\right)=\left[F\left(0\right)-F_{e}\right]\;e^{-t/T_{L}}+F_{e}$ (dashed lines). The red crash symbols indicate that the drone crashed into the tree, and the final target position is marked by green target symbols. Comparison of average (**D**) learning time ($T_{L}$), (**E**) equilibrium objective function ($F_{e}$), (**F**) adaptability to the changing environment, and (**G**) computing power consumption of the synstor circuit, human operators, and ANN in multiple trials. The adaptability to changing environment is evaluated by the successful rate of learning how to drive the drone to its final target without collisions in multiple trials. The power consumption of the synstor circuit is for concurrent inference and learning, whereas the power consumption of the ANN is the aggregate of the power consumptions for sequential inference and learning.

The synstor circuit, human operators, and ANN successfully learned to drive the drone toward its target position across the mountain area without trees or strong wing while minimizing the objective function $F=\frac{1}{2}{\left(s-\hat{s}\right)}^{2}$. The F−t curves can be best fitted by $F\left(t\right)=\left[F\left(0\right)-F_{e}\right]\;e^{-t/T_{L}}+F_{e}$ (Materials and Methods, Eq. 6, and Fig. 6) to extrapolate the average learning time $T_{L}$ and the equilibrium objective function $F_{e}$ when $t\gg T_{L}$ and $d\bar{F}/\mathrm{dt}\approx 0$, which represents the performance of the drone when W has been modified to $\hat{W}$. The average $T_{L}$ (4.4 s) of the synstor circuit in multiple trials was shorter than the average $T_{L}$ (6.6 s) of the human operators and significantly superior to the average $T_{L}$ (127,568 s) of the ANN in multiple trials (Fig. 6D). The drone performance, represented by $F_{e}$, of the drone driven by the synstor circuit [0.10 arbitrary unit (a.u.)] and humans (0.25 a.u.) is significantly superior to that of the drone driven by the ANN (0.44 a.u.) in their multiple trials (Fig. 6E). When the drone entered the environment with trees and strong wind, only the synstor circuit and most human operators were able to navigate the drone to its final target position, demonstrating their ability to adapt to the environment with the gale and avoid collisions with the trees; in contrast, the ANN consistently failed to adapt to the complex environment with trees and gale, resulting in collisions in multiple trials (Fig. 6, A to C, and movie S1). The adaptability to the changing environment is evaluated by the success rate of learning to drive the drone to its final target without collisions in multiple trials. The adaptability (100%) of the synstor circuit is better than that (64%) of the human operators and significantly superior to that (0%) of the ANN (Fig. 6F). The power consumption of the synstor circuit (158 nW; Materials and Methods) for the concurrent execution of the inference and learning algorithms was also seven orders of magnitude lower than the aggregate power consumption of the computer (6.3 W; Materials and Methods) executing the learning and inference algorithms sequentially (Fig. 6G). Estimating the power consumption of the human brain for the inference and learning is difficult.

## DISCUSSION

We introduced an intelligent system based on a synstor circuit with concurrent real-time inference (I=Wx; Eq. 1) and learning ($\overset{\cdot }{W}=\alpha \;z⊗x$; Eq. 2) functionality emulating the brain. Theoretical analysis indicates that the simultaneous execution of inference and learning can minimize the objective function $F=\frac{1}{2}{\left(s-\hat{s}\right)}^{2}$ of the system, tuning the system states (s) toward their target states ($\hat{s}$) by modifying W toward $\hat{W}=\text{arg}\;\underset{W}{\text{min}}F$. A circuit of three-terminal synstors was fabricated by integrating a heterojunction of a Si channel/SiO_2_ dielectric layer/defect-reduced ferroelectric Hf_0.5_Zr_0.5_O_2_ layer/defect-enhanced WO_2.8_ reference electrode vertically, and Schottky contacts between the Si channel/TiSi_0.9_/Ti input and output electrodes laterally in each synstor. Paired voltage pulses ($x_{m}$ and $z_{n}$) applied on the input and output electrodes of a synstor with respect to a grounded reference electrode progressively modify the individual ferroelectric domains within the Hf_0.5_Zr_0.5_O_2_ layer, thereby adjusting the synstor conductance in analog mode based on the learning function ($\overset{\cdot }{W}=\alpha \;z⊗x$); a single voltage pulse ($x_{m}$) applied to the input electrode of a synstor induces a current through the synstor channel according to the inference function (I=Wx). The voltage primarily drops laterally across the Schottky junction and does not modify the ferroelectric domains within the Hf_0.5_Zr_0.5_O_2_ layer or the synstor conductance (i.e., ${\overset{\cdot }{w}}_{\mathrm{nm}}=0$ when $x_{m}\neq 0$ and $z_{n}=0$) as per the learning rule ($\overset{\cdot }{W}=\alpha \;z⊗x$). Compared to other neuromorphic devices like two-terminal memristors and ferroelectric devices, which can only perform inference and learning sequentially, the synstor circuit uniquely enables concurrent execution of inference and learning, allowing the circuit to operate in a Super-Turing mode (Table 1). The defect-reduced Hf_0.5_Zr_0.5_O_2_ layer with multiple switchable ferroelectric domains effectively improves the device performance and facilitates the precise, reliable, fast, and repetitive modification of synstor conductance with 1000 analog conductance levels within a conductance range of 0 to 60 nS, a learning accuracy of 36 pS, 1.6 × 10^11^ repetitive learning cycles, and a rapid modification time within 10 ns. In experiments aimed at navigating a drone toward target positions while avoiding obstacles in a simulated complex aerodynamic environment with time-varying wind, without any prior learning, a synstor circuit and human operators executed real-time learning and inference concurrently, in comparison with an ANN run on a computer executing inference and learning sequentially in Turing mode. During concurrent real-time inference and learning in a synstor circuit, the conductance ($w_{\mathrm{nm}}$) of each synstor can be dynamically adjusted based on signals ($x_{m}$ and $z_{n}$) received from the input and output electrodes connected by the synstor in parallel analog mode. The learning process aims to optimize the objective function (F) in response to environmental changes in real time. In contrast, during sequential inference and learning processes in the ANN or other neuromorphic resistor circuits, their weight matrixes cannot be adjusted during inference according to real-time environmental changes. The optimal weight matrices were derived during iterative learning processes with serial statistical evaluation and modification of numerous various combinations of W elements. The experimental learning times ($T_{L}$) for both the synstor circuit and human operators was four orders of magnitude shorter than those of the ANN. The ANN consistently was unable to adapt to the complex environment with trees and strong variable winds, resulting in collisions in every trials. In contrast, the synstor circuit and most human operators successfully adapted to the environment, driving the drone to its final target without collisions. As evaluated by the equilibrium objective function ($F_{e}$) when W has been modified to $\hat{W}$, the performance of the drone driven by the synstor circuit and human operators was significantly superior to that of the drone driven by the ANN. A single-layer synstor circuit can execute learning and inference concurrently in real time, dynamically optimizing the W matrix, triggering optimal output actuation signals (y) to minimize the objective function (F). Conversely, during the sequential inference and learning processes, the ANN expended large amounts of energy and time to store the weight matrix, x, and y data, execute the learning functions in its computing units, and transfer the data back and forth between different circuits (*4*–*7*, *12*, *13*, *15*, *17*–*19*, *22*). The conductance of the synstors (<60 nS) was significantly lower than that of transistors (~0.1 mS) (*5*–*7*, *48*), memristors (~1 μS to 10 mS) (*15*, *17*, *49*–*52*), and phase change memory resistors (1 μS to 10 mS) (*22*) (fig. S11). Consequently, the power consumption of synstors is significantly lower than that of other neuromorphic devices (Table 1). The power consumption of the synstor and neuron circuits (158 nW) for real-time concurrent inference and learning was seven orders of magnitude lower than the aggregate power consumption (6.3 W) of the computer executing the learning and inference functions sequentially in the ANN. The computing energy efficiency of the synstor circuit is calculated (Materials and Methods) as 1.2 × 10^17^ operations per second per watt (OPS/W), which is significantly higher than those of other neuromorphic devices. However, the area of the microscale synstor (2 × 10^−4^ mm^2^) is considerably larger than those of nanoscale neuromorphic devices, leading to a much lower area efficiency (1.5 × 10^12^ OPS/mm^2^; Materials and Methods) compared with nanoscale neuromorphic devices (Table 1). Scaling down the synstors could further reduce their conductance, power consumption, and area, thereby enhancing both energy and area efficiency. On the basis of simulations (Materials and Methods), a HfZrO-based synstor can be scaled down to a channel length of 100 nm (fig. S12), and a HfZrO-based synstor crossbar circuit can be scaled up to 10^8^ synstors with 10^4^ input and output channels using the nanoscale fabrication techniques of the HfZrO-based ferroelectric transistor circuit (*40*, *53*). Synstor circuits offer a brain-inspired Super-Turing computing platform for AI systems with extremely low power consumption, high-speed real-time learning and inference, and agile adaptability to dynamic complex environments.

**Table 1.** Comparison of devices. Comparative analysis of synstors (this work) alongside biological synapses (*28*), ferroelectric transistors (*38*, *40*, *41*, *53*), and memristors (*17*, *18*, *49*, *51*).

	Synstor (this work)	Synapse	Ferroelectric transistor	Memristor
Learning and inference signal magnitudes	Same	Same	Different	Different
Concurrent learning and inference	Yes	Yes	No	No
Turing mode	Yes	Yes	Yes	Yes
Super-Turing mode	Yes	Yes	No	No
Real-time adaptability	Yes	Yes	No	No
Real-time error correction	Yes	Yes	No	No
Conductance (S)	<6 × 10^−8^	~10^−11^–10^−9^	<10^−5^	<10^−4^
Power consumption (W)	2.5 × 10^−9^	~10^−13^–10^−11^	~10^−6^	~4 × 10^−6^
Energy efficiency (OPS/W)	1.2 × 10^17^	~10^15^–10^17^	5.7 × 10^13^	1.9 × 10^14^
Device area (mm^2^)	2 × 10^−4^		3.6 × 10^−8^	2.5 × 10^−7^
Area efficiency (OPS/mm^2^)	1.5 × 10^12^		1.6 × 10^15^	7.2 × 10^12^
Endurance (switching cycles)	>10^11^		>10^8^	>10^12^

## MATERIALS AND METHODS

### Theoretical analysis of a synstor circuit

The temporal mean of the **z** voltage pulses applied on the output electrode of the synstor circuit satisfies

$$\bar{z}=0$$ (3)

and the covariance between *z*_*n*_ and *y*_*n*′_

$$\bar{z_{n}y_{n\prime }}=\eta_{n}\;\delta_{\mathrm{nn}\prime }$$ (4)

where δ_*nn*′_ denotes the Kronecker delta with $\delta_{\mathrm{nn}\prime }=\left\{ \begin{array}{c} 0\;\text{when}\;n\prime \neq n\\ 1\;\text{when}\;n\prime =n \end{array}\right.$, and $\eta_{n}$ denotes a parameter. The covariance $\bar{z_{n}y_{n}}$ is defined as the mean of the product of the deviations of $z_{n}$ and $y_{n}$ from their individual means ${\bar{z}}_{n}$ and ${\bar{y}}_{n}$. $\bar{z_{n}y_{n\prime }}=\bar{\left(z_{n}-{\bar{z}}_{n}\right)\left(y_{n\prime }-{\bar{y}}_{n\prime }\right)}=\bar{z_{n}\left(y_{n\prime }-{\bar{y}}_{n\prime }\right)}$, where ${\bar{z}}_{n}=0$ (Eq. 3) and $\bar{z_{n}{\bar{y}}_{n\prime }}={\bar{z}}_{n}{\bar{y}}_{n\prime }=0$. The learning rule observed in synapses within neurobiological circuits, known as STDP (*28*, *30*), can also be formulated as $\frac{dW}{\mathrm{dt}}=\alpha \;z\left(t\right)⊗x\left(t\right)$ (Eq. 2) with $z_{n}\left(t\right)=\left\{\begin{array}{c} A_{-}e^{\left(t-t_{n}^{y}\right)/\tau_{-}}\;\text{when}\;t<t_{n}^{y}\\ -A_{+}e^{-\left(t-t_{n}^{y}\right)/\tau_{+}}\;\text{when}\;t\geq t_{n}^{y} \end{array}\right.$, where $t_{n}^{y}$ denotes the moment when a pulse (y) is triggered at the *n*th postsynaptic neuron, $A_{+}>0$ and $A_{-}>0$ denote amplitude constants, and $\tau_{+}>0$ and $\tau_{-}>0$ denote time constants. In STDP, z fulfills the conditions $\bar{z}=0$ (Eq. 3) and $\bar{z_{n}y_{n\prime }}=\eta_{n}\;\delta_{\mathrm{nn}\prime }$ (Eq. 4) with $\eta_{n}\geq 0$ for STDP and $\eta_{n}\leq 0$ for anti-STDP (*30*). The change rate of the output current ($I_{n}$) due to learning, ${\left(\frac{\partial I_{n}}{\partial t}\right)}_{L}=\sum_{m}\frac{\partial I_{n}}{\partial w_{\mathrm{nm}}}{\overset{\cdot }{w}}_{\mathrm{nm}}=\sum_{m}x_{m}\left(\alpha z_{n}x_{m}\right)=2|\alpha |z_{n}F_{x}$, where $\frac{\partial I_{n}}{\partial w_{\mathrm{nm}}}=x_{m}$ due to $I_{n}=\sum_{m}w_{\mathrm{nm}}x_{m}$ (Eq. 1), ${\overset{\cdot }{w}}_{\mathrm{nm}}=\alpha z_{n}x_{m}$ (Eq. 2), and $F_{x}=\frac{1}{2}\sum_{m}x_{m}^{2}$. Although it is unfeasible to predefine a mathematical model for the correlation between $F_{x}$ and $y_{n}$ that is dynamically changing, a linear model can be constructed to describe their relationship during a learning period, $F_{x}=\sum_{n}{\left(\frac{\partial F_{x}}{\partial y_{n}}\right)}_{L}\;y_{n}+F_{x}^{0}$. In the model, $\frac{\partial F_{x}}{\partial y_{n}}={\left(\frac{\partial F_{x}}{\partial y_{n}}\right)}_{L}$ denotes the regression coefficient during the learning period, and $F_{x}^{0}$ represents the portion of $F_{x}$ that is not correlated with y or z. On the basis of the linear model, $\bar{z_{n}F_{x}}=\sum_{n\prime }{\left(\frac{\partial F_{x}}{\partial y_{n\prime }}\right)}_{L}\bar{z_{n}y_{n\prime }}+\bar{z_{n}F_{x}^{0}}={\left(\frac{\partial F_{x}}{\partial y_{n}}\right)}_{L}\bar{z_{n}y_{n}}$, where $\bar{z_{n}F_{x}^{0}}={\bar{z}}_{n}\bar{F_{x}^{0}}=0$, and $\bar{z_{n}y_{n\prime }}=0$ for n′≠n (Eq. 4). The average change rate of $I_{n}$ due to learning during the learning period ${\bar{\left(\frac{\partial I_{n}}{\partial t}\right)}}_{L}=2|\alpha |\bar{z_{n}F_{x}}=2|\alpha |{\left(\frac{\partial F_{x}}{\partial y_{n}}\right)}_{L}\bar{z_{n}y_{n}}$, where $\bar{z_{n}F_{x}}$ is substituted by ${\left(\frac{\partial F_{x}}{\partial y_{n}}\right)}_{L}\bar{z_{n}y_{n}}$. On the basis of the linear model, the average change rate of the objective function $F=\frac{1}{2}{\left(s-\hat{s}\right)}^{2}$ due to learning during the learning period ${\bar{\left(\frac{\partial F}{\partial t}\right)}}_{L}=\bar{\frac{\partial F}{\partial F_{x}}\sum_{n}\frac{\partial F_{x}}{\partial y_{n}}\frac{\partial y_{n}}{\partial I_{n}}{\left(\frac{\partial I_{n}}{\partial t}\right)}_{L}}={\left(\frac{\partial F}{\partial F_{x}}\right)}_{L}{\left(\frac{\partial F_{x}}{\partial y_{n}}\right)}_{L}{\left(\frac{\partial y_{n}}{\partial I_{n}}\right)}_{L}{\bar{\left(\frac{\partial I_{n}}{\partial t}\right)}}_{L}=2|\alpha |{\left(\frac{\partial F}{\partial F_{x}}\right)}_{L}\sum_{n}{\left(\frac{\partial F_{x}}{\partial y_{n}}\right)}_{L}^{2}{\left(\frac{\partial y_{n}}{\partial I_{n}}\right)}_{L}\bar{z_{n}y_{n}}$, where $\frac{\partial F}{\partial F_{x}}={\left(\frac{\partial F}{\partial F_{x}}\right)}_{L}$ denotes the regression coefficient in the linear model $F={\left(\frac{\partial F}{\partial F_{x}}\right)}_{L}F_{x}+F^{0}$ with $F^{0}$ as the portion of F that is not correlated with $F_{x}$, $\frac{\partial y_{n}}{\partial I_{n}}={\left(\frac{\partial y_{n}}{\partial I_{n}}\right)}_{L}$ denotes the regression coefficient in the linear model $y_{n}={\left(\frac{\partial y_{n}}{\partial I_{n}}\right)}_{L}I_{n}+I_{n}^{0}$ with $I_{n}^{0}$ as the portion of $I_{n}$ that is not correlated with $y_{n}$, and ${\bar{\left(\frac{\partial I_{n}}{\partial t}\right)}}_{L}$ is substituted by $2|\alpha |{\left(\frac{\partial F_{x}}{\partial y_{n}}\right)}_{L}\bar{z_{n}y_{n}}$. F is a monotonically increasing function of $F_{x}$; thus, ${\left(\frac{\partial F}{\partial F_{x}}\right)}_{L}\geq 0$. $y_{n}$ is a monotonically increasing function of $I_{n}$; thus, ${\left(\frac{\partial y_{n}}{\partial I_{n}}\right)}_{L}\geq 0$. ${\bar{\left(\frac{\partial F}{\partial t}\right)}}_{L}=2|\alpha |{\left(\frac{\partial F}{\partial F_{x}}\right)}_{L}\sum_{n}{\left(\frac{\partial F_{x}}{\partial y_{n}}\right)}_{L}^{2}{\left(\frac{\partial y_{n}}{\partial I_{n}}\right)}_{L}\bar{z_{n}y_{n}}$ with $2|\alpha |{\left(\frac{\partial F}{\partial F_{x}}\right)}_{L}{\left(\frac{\partial F_{x}}{\partial y_{n}}\right)}_{L}^{2}{\left(\frac{\partial y_{n}}{\partial I_{n}}\right)}_{L}\geq 0$ and $\bar{z_{n}y_{n}}\leq 0$ (Eq. 4); therefore, ${\bar{\left(\frac{\partial F}{\partial t}\right)}}_{L}\leq 0$. The average change rate of the objective function $\bar{\left(\frac{\mathrm{dF}}{\mathrm{dt}}\right)}={\bar{\left(\frac{\partial F}{\partial t}\right)}}_{L}+{\bar{\left(\frac{\partial F}{\partial t}\right)}}_{0}$ with ${\bar{\left(\frac{\partial F}{\partial t}\right)}}_{0}$ as the average change rate of F not related to learning (i.e., without W modification). When $\text{s}=\hat{s}$, $F=\frac{1}{2}{\left(s-\hat{s}\right)}^{2}=0$, x=0, $\overset{\cdot }{W}=\alpha \;z⊗x=0$ (Eq. 2), and W reaches the optimal steady value $\hat{W}=\text{arg}\;\underset{W}{\text{min}}F$. When $W\neq \hat{W}$, $s\neq \hat{s}$; thus, $F=\frac{1}{2}{\left(s-\hat{s}\right)}^{2}>0$. When ${\bar{\left(\frac{\partial F}{\partial t}\right)}}_{L}\leq -{\bar{\left(\frac{\partial F}{\partial t}\right)}}_{0}\leq 0$

$$\bar{\left(\frac{\mathrm{dF}}{\mathrm{dt}}\right)}\leq 0$$ (5)

$\bar{F}$ represents a Lyapunov function. When $\bar{\left(\frac{\mathrm{dF}}{\mathrm{dt}}\right)}<0$, $\bar{F}$ is asymptotically decreased, thus resulting in modification of W toward $\hat{W}=\text{arg}\;\underset{W}{\text{min}}F$ during concurrent learning and inference processes.

The concurrent execution of the inference (Eq. 1) and learning (Eq. 2) in the synstor circuit tends to minimize the average objective function $\bar{F}$ over time. When the covariance between z and x, $\bar{z⊗x}=0$, then $\bar{\frac{dW}{\mathrm{dt}}}=0$, $\bar{\left(\frac{\mathrm{dF}}{\mathrm{dt}}\right)}=0$, and $W=\hat{W}=\text{arg}\;\underset{W}{\text{min}}F$ remain unchanged during the execution of the inference function, $I=\hat{W}x$.

### Synstor circuit fabrication process

The fabrication process of a synstor circuit is depicted in fig. S1. The synstor circuit was fabricated on a silicon-on-insulator chip with a 220-nm-thick p-type Si layer with a boron doping concentration of 10^15^/cm^3^ on a 3-μm-thick buried silicon oxide layer on a 725-μm-thick Si wafer. A photoresist layer was patterned on the Si surface as an etching mask by ultraviolet (UV) photolithography. As shown in fig. S1A, a 5-μm-wide Si channel was made by reactive ion etching (RIE; Oxford Plasmalab 80 Plus RIE). The photoresist was stripped with acetone, isopropanol, and deionized water. The wafer was cleaned using the standard Radio Corporation of America (RCA) cleaning process. As shown in fig. S1B, the surface of the Si channel was oxidized in a thermal oxidation furnace at 900°C in O_2_ for 10 s to grow a 3.5-nm-thick SiO_2_ layer. A photoresist layer was patterned by UV photolithography on the surface of the SiO_2_ layer, and the photoresist patterns were used as an etching mask to etch the SiO_2_ layer by RIE (Oxford Plasmalab 80 Plus RIE) in the contact areas for input/output electrodes (fig. S1C). A 300-nm-thick Ti layer was deposited by electron beam evaporation (CHA Industries Mark 40), and Ti input/output electrodes in the contact areas were made by lifting off a prepatterned photoresist layer (fig. S1D). A 40-nm-thick Al_2_O_3_ sacrificial layer was grown on the chip by atomic layer deposition (ALD; Fiji Ultratech ALD). The chip was annealed in forming gas (5% H_2_ in N_2_) at 460°C for 30 min to form a titanium silicide layer sandwiched between the Si channel and Ti input/output electrodes (fig. S1E). The Al_2_O_3_ sacrificial layer protected the materials from undesirable oxidation during the annealing. After the annealing, the sacrificial layer was selectively etched away with a 3% tetramethylammonium hydroxide aqueous solution. A 12.6-nm-thick Hf_0.5_Zr_0.5_O_2_ layer was deposited on the chip by ALD (Fiji Ultratech ALD) at 200°C using tetrakis(dimethylamino)hafnium(IV) and tetrakis(dimethylamino)zirconium(IV) precursors (fig. S1F). An 80-nm-thick W oxide layer was deposited on the Hf_0.5_Zr_0.5_O_2_ layer by magnetron sputtering (Denton Discovery), and W oxide reference electrodes were patterned by lifting off a photoresist layer (fig. S1G). The chip was then annealed in a rapid thermal annealing system (Modular Process Technology RTP-600xp) at 500°C for 1 min to crystalize and induce a heterojunction between ferroelectric Hf_0.5_Zr_0.5_O_2_ layer and a WO_2.8_ reference electrode. Last, the Hf_0.5_Zr_0.5_O_2_ layer on the contact pads of the input/output electrodes was etched away using photolithography and RIE (Oxford Plasmalab 80 Plus RIE).

### Material characterization and electrical tests of synstors

Structural and material composition profiles of the synstor chip were characterized using STEM, EDX, and EELS analysis. EDX analysis was performed using JEOL JEM-2800 TEM operated at 200 kV. Atomic-resolution STEM and EELS analysis was performed with a JEOL Grand ARM TEM operated at 300 kV with a spherical aberration corrector. During the electric tests, the reference electrodes of the synstors and control devices were always grounded. Current-voltage characteristics were measured with a Keithley 4200 semiconductor parameter analyzer. The electrical voltage pulses applied to the input and output electrodes of the devices and circuits were generated by a field-programmable gate array (FPGA; National Instruments, cRIO-9063), computer-controlled modules (National Instruments, NI-9264), and a Tektronix AFG3152C waveform/function generator. Currents flowing through the synstors were measured by a semiconductor parameter analyzer, computer-controlled circuit modules (National Instruments, NI-9205 and NI-9403), and oscilloscope (Tektronix TDS 3054B). Testing protocols were programmed (NI LabVIEW) and implemented in an embedded FPGA (Xilinx), a microcontroller, and a reconfigurable Input/Output interface (NI CompactRIO).

### Analog modification and uniformity tests of synstors

To examine the analog modification and uniformity of the circuit, the 64 synstors in the circuit were tuned to 1000 targeted analog values, $w_{t}$, evenly separated by $\Delta w_{t}=59\;\text{pS}$, by applying a train of paired 10-μs-wide *x* and *z* pulses concurrently with an amplitude of −4 V (or 4 V) on each synstor until their conductance values reached the targeted analog values closely.

### Endurance tests of the synstors

The endurance testing of the synstors was performed by modification of their conductance to high (16 nS) and low (5 nS) conductance values by iterative application of paired 3-μs-wide *x* and *z* pulses concurrently with an amplitude of −6 V (or 7 V). Endurance testing of the synstors was also conducted by adjusting their conductance to the maximum (60 nS) and minimum (0.6 nS) values through iterative application of paired 200-μs-wide *x* and *z* pulses concurrently with an amplitude of −6 V (or 7 V).

### Synstor test at different temperatures

The currents (*I*) through synstors were measured as a function of input voltage (*x*) using a Keithley 4200 semiconductor parameter analyzer. The synstors were tested at temperatures between 24° and 80°C. Conductance was adjusted to a target value by iteratively applying paired 3-μs-wide *x* and *z* pulses concurrently with amplitudes of −6 or 7 V.

### Resistance tests of WO_2.8_ reference electrodes

WO_2.8_ thin-film resistors, with a thickness of 80 nm, a width of 40 μm, and a length of 100 μm, were fabricated on SiO_2_ surface using the methods described in the synstor fabrication process. The current-voltage characteristics and resistance of the WO_2.8_ resistor were measured using a Keithley 4200 semiconductor parameter analyzer over a temperature range of 80 to 320 K in a Lake Shore cryogenic probe station.

### Nonvolatile memory tests of the synstors

Synstor conductance values were modified to different initial analog conductance values, *w*(0), by applying paired positive or negative pulses, and their conductance values, *w*, were measured versus time, t, over 1.2 × 10^6^ s at a temperature of 40° and 85°C, respectively.

### Polarization-voltage (*P*-*V*) measurement on HfZrO films

We fabricated capacitors composed of a HfZrO film for its *P*-*V* measurement. A blanket thin film of 70-nm-thick W was sputter coated as a bottom electrode (BE) on a 300-nm-thick SiO_2_ layer grown thermally on a silicon wafer. A 10-nm-thick HfZrO film was deposited on the BE via ALD as described in Materials and Methods about synstor fabrication. A 45-nm-thick W layer was then sputter deposited as a top electrode, and a 50-nm-thick platinum (Pt) was used to cap off the capacitors. Capacitors with a 30 μm–by–30 μm W top electrode patterned via photolithography and liftoff processes were annealed at 500°C.

### Drone flying in a simulated environment

The experiments were performed by flying a drone in an environment (Fig. 5 and movie S1) simulated by running an AirSim program (Microsoft Co.) in a Dell computer with an Intel i9-12900 CPU and NVIDIA RTX 3060 Ti GPU. During the flight, wind randomly changed its direction and speed from 0 to 17.5 m/s (fig. S8). The positions of the drone and target were determined by a simulated global positioning system. To avoid collisions with obstacles such as mountains and trees in the environment, the depth profiles of the obstacles were detected by a simulated camera mounted on the drone and analyzed to identify local flight target positions with obstacle clearance and minimal objective function $F=\frac{1}{2}{\left(s-\hat{s}\right)}^{2}$, where $s-\hat{\text{s}}$ as the deviations of the drone position (s) from its target position ($\hat{s}$) along upward, downward, forward, backward, left, right, clockwise, and anticlockwise (yaw) dimensions (fig. S7D).

### Experiments to drive a drone by a synstor circuit

The conductance values in the matrix of the synstor circuit, W, were set to random values before each learning experiments started (fig. S9). As shown in Fig. 5, figs. S7A and S8A, and movie S1, $s-\hat{s}$ was converted to voltage pulses, **x**, with an amplitude of −3 or 4.2 V and a duration of 10 ns, which were input to the synstor circuit by the interface circuit (FPGA, Xilinx, Kintex-7). The firing rate of **x** pulses increased monotonically with increasing $s-\hat{s}$. **x** generated currents, **I** = **Wx** (Eq. 1), via the synstor circuit. The currents, **I**, flowed through the synstors to interface circuits and triggered the actuation pulses, y, to drive the drone, and the voltage pulses, **z**, applied on the output electrodes of the synstor circuit to modify the synstor conductance matrix, **W**, by following the learning rule, $\overset{\cdot }{W}=\alpha \;z⊗x$ (Eq. 2). Neuron circuits were designed in our laboratory and fabricated in Taiwan Semiconductor Manufacturing Company to emulate the functions of biological neurons. When $I<I_{\mathrm{th}}\approx 30\;\text{nA}$, the threshold current of the neuron circuit, no actuation pulse was triggered. When $I_{\mathrm{th}}\leq I\leq I_{S}\approx 60\;\text{nA}$, the saturation current of the neuron circuit, the average firing rate of the actuation pulses increased monotonically with increasing I. When $I>I_{S}$, the average firing rate of the actuation pulses was saturated at ∼14 Hz. The output pulses from the neuron circuits were converted to actuation signals, y, by the interface circuit (FPGA, Xilinx, Kintex-7) to drive the drone (Fig. 5 and fig. S7A). The voltage pulses, z, were triggered from the neuron circuits. The z pulses satisfied the conditions ${\bar{z}}_{n}=0$ (Eq. 3) and $\bar{z_{n}y_{n}}\leq 0$ (Eq. 4). When a $y_{n}$ pulse was triggered at moment $t=t_{n}$ from the *n*th output neuron of the synstor circuit, a train of negative (or positive) $z_{n}$ pulses were triggered at the *n*th (or *n*th complimentary) output electrodes within the time window $t_{n}<t<t_{n}+t_{w}$, and a train of positive (or negative) $z_{n}$ pulses were triggered at the *n*th (or *n*th complimentary) output electrodes within the time window $t_{n}+\tau_{+}<t<t_{n}+\tau_{+}+t_{w}$, where τ_+_ = 1180 ms and *t*_*w*_ = 40 ms represents the duration of the time window of the pulse trains.

### Experiments to navigate a drone by human operators

In the experiments with the drone driven by humans under the same environment of the synstor circuit experiments (Materials and Methods, Fig. 5, figs. S7B and S8B, and movie S1), 14 human operators without any prior knowledge of the drone and its control system visually received $s_{L}$ signals displayed on a computer monitor and were instructed to drive the drone toward its local target position and avoid the collision with obstacles by minimizing the $s-\hat{s}$ values. The $s-\hat{s}$ signals were processed by the neurobiological circuits in the human brains, triggering actuation pulses y to drive the drone by pressing eight keys in a keyboard (fig. S8B). The firing rates of y pulses were proportional to the keystroke times.

### Experiments to drive a drone by an ANN running on a computer

The experiments with the drone driven by a computer-based ANN were performed under the same environment as the synstor circuit and human experiments (Fig. 5, figs. S7C and S8C, and movie S1). Before the learning experiment started, the digital synaptic weight matrix, W, in the ANN was also set to random values, the same as for the trials of the synstor circuit. The time required to execute learning function was significantly longer than that to execute the inference function during the flight (fig. S10F); thus, it is infeasible to execute the real-time learning on the computer, and the inference and learning are executed sequentially. The ANN running on a Dell computer (Intel i9-12900 CPU and NVIDIA RTX 3060 Ti GPU) received the $s-\hat{s}$ signals, converted $s-\hat{s}$ to input signals, x, and induced output current I=W(n) x, triggering actuation pulses, y, to modify $s_{L}$ at *n*th episode to drive the drone for ∼30 s (Fig. 5C). After the *n*th flight episode ended, the saved data, $s-\hat{s}$, y, and W(n) from the *n*th episode were sent back to the computer to execute a reinforcement learning function (*47*) (Supplementary Materials) for ∼550 s to modify W(n) to W(n+1), which reset the ANN to drive the drone at the (*n* + 1)th flight episode iteratively until the drone crashed into an obstacle or reached the final target. ANNs with (i) 8 neurons at its input layer, no hidden layer, and 9 neurons at its output layer interconnected by 72 synapses and (ii) 8 neurons at its input layer, 128 neurons at its hidden layer, and 9 neurons at its output layer interconnected by 2176 synapses were tested to drive the drone toward a local target position in the learning experiments with a learning rate of 10^−8^, 2 × 10^−8^, 5 × 10^−8^, and 10^−^, respectively (fig. S10). The optimal ANN with no hidden layer and a learning rate of 5 × 10^−8^ had the shortest learning time (fig. S10I) and were then used in the learning experiments to drive the drone toward the final target.

### Average learning time

In the learning processes of synstor circuits, humans, and ANN, the change rate of the objective function, $\overset{\cdot }{F}$, is a nonlinear function of F but can be best fitted by a linear dynamic model $\overset{\cdot }{F}=-\left(F-F_{e}\right)/T_{L}$ and its solution over the learning processes (Fig. 6)

$$F\left(t\right)=\left[F\left(0\right)-F_{e}\right]\;e^{-t/T_{L}}+F_{e}$$ (6)

where the fitting parameter $T_{L}$ represents the average initial learning time, and $F_{e}$ represents the quasiequilibrium objective function when $t\gg T_{L}$ and $d\bar{F}/\mathrm{dt}\approx 0$.

### Power consumptions of concurrent inference and learning by the synstor circuit

When voltage pulses were applied on a 8 × 8 crossbar synstor circuit, the average power consumption of the circuit (excluding the power consumptions of interface circuits), $P=I⊗x=\left(\text{wx}\right)⊗x\approx w_{T}\;V_{a}^{2}D_{p}$, where $w_{T}$ denotes the total conductance of the synstors in the circuit, $V_{a}$ denotes the magnitude of pulses, and $D_{p}$ denotes the average duty cycle of the pulses. During the concurrent signal processing and learning in the synstor circuit for the drone, $w_{T}\approx 10\;\text{nS}$, $V_{a}=4.2\;V$, and $D_{p}=0.014$; thus, P≈158 nW. On the basis of the circuit simulation, the total power consumption of the eight neuron circuits was ~0.78 nW.

### Computing energy and area efficiency of the synstor circuit

A crossbar synstor circuit with an M×N synstors executes the inference algorithm with a computing speed of $2\mathrm{MN}f_{i}$, which corresponds to MN analog multiplications between W and x, and MN analog accumulations, performed at an analog signal input rate, $f_{i}$. The learning algorithm is executed at a computing speed of $4\mathrm{MN}f_{i}$, which corresponds to 3MN analog outer products between α, x, and z, and MN modifications of W, also at the rate of $f_{i}$. The average computing speed for a synstor circuit with M×N synstors to concurrently execute the inference and learning algorithms in analog parallel mode is $V_{c}=6\mathrm{MN}f_{i}$. For $f_{i}=50\;\text{MHz}$, the computing speed of the 8×8 crossbar synstor circuit is $V_{c}=1.92\times 10^{10}\;\text{OPS}$. The computing energy efficiency is calculated as $E_{f}=V_{c}/P\approx 1.2\times 10^{17}\;\text{OPS}/W$. With a synstor active area of approximately $S\approx 2\times 10^{-4}\;{\text{mm}}^{2}$, the average area efficiency of the 8×8 crossbar synstor circuit is $E_{s}=V_{c}/\left(\mathrm{MNS}\right)\approx 1.5\times 10^{12}\;\text{OPS}/{\text{mm}}^{2}$.

### Power consumptions of sequential inference and learning by the computer

According to analysis from Python toolkits “keras_flops” and “pyperf,” the speeds for sequentially executing the inference and learning programs on the computer were estimated to be 3.9 kilo floating-point operations per s and 2.0 giga floating-point operations per s, respectively. With a computing energy efficiency of 3.1 giga floating-point operations per s (*54*), the power consumption for sequentially executing the inference and learning programs on the computer was 12 μW and 6.3 W, respectively. The aggregate power consumption of the ANN for sequential inference and learning is 6.3 W (Fig. 6).

### Computer-aided device and circuit simulation

On the basis of the properties of synstors and their circuits, we have designed and simulated synstors and their circuits by SPICE (Simulation Program with Integrated Circuit Emphasis) simulators (SPECTRE from Cadence and HSPICE from Synopsys) (*35*). The simulator performed numerical calculations of the device physics by solving Poisson’s equation describing the electrostatics and drift-diffusion carrier transport under a set of boundary conditions defined by the device structure. Quasistationary simulations were conducted under various voltage biases on the input/output electrodes of the synstors with respect to the grounded reference electrodes. The band diagrams of the synstors were extracted from the simulations, and the electronic properties of the synstors were analyzed by the simulations. According to the Technology Computer-Aided Design simulation, when the synstor channel length and the reference electrode length are scaled down to 100 and 40 nm, respectively, the synstor still functions properly for inference and learning (fig. S12). We have designed and simulated a synstor circuit based on a GlobalFoundries 28-nm ferroelectric process, which has an average synstor conductance 〈w〉≈0.2 nS, $R_{e}\approx 0.54\;\text{ohms}$; thus, $M,N≲10^{4}$, i.e., a single crossbar synstor circuit can be scaled up to $M,N=10^{4}$ with $M\cdot N=10^{8}$ synstors.

### Sequential inference and reinforce learning executed in the ANN

The sequential inference (flight) and reinforcement learning functions (*47*) executed in the ANN is briefly described mathematically as follows:

Initialize random synaptic weight matrix W(0) in the ANN.

Loop for episodes $n=1,2,\ldots ,N_{T}$ until the drone reaches the final target or crashes into an obstacle.

In the *n*th inference (flight) episode that lasted ~30 s, generate data $s-\hat{s}$, y, and W(n) by following the inference function $f\left[y\left(t\right)|s_{L}\left(t\right),W\left(n\right)\right]$ in the ANN at discrete time steps $t=0,1,\ldots ,N_{D}$ at the frequency of 12.5 kHz with input signals, $s-\hat{s}$, as the deviations of the drone position from the local target W(n) as the ANN synaptic weight matrixes in the *n*th flight episode and y(t) as actuation pulses to drive the drone.

After the *n*th inference (flight) episode ends, start the *n*th learning episode.

In the *n*th learning episode, loop for discrete time steps *t* = 0, 1, …, *N*_*D*_, $\Gamma \left(t+1\right)=\Gamma \left(t\right)+r_{L}\mu^{t}\left(G\left(t\right)-b\right)\nabla_{\Gamma }\text{ln}\left\{f,\left[y\left(t\right)|\left(s-\hat{s}\right),\Gamma_{k}\right]\right\}$, where Γ(0)=W(n), learning rate $r_{L}>0$, discount factor μ > 0, $G\left(t\right)=\sum_{j=t+1}^{N_{D}}\mu^{j-t-1}R\left(j\right)$, baseline $b=\sum_{t}G\left(t\right)/N_{D}$, and reward R(j).

Update $W\left(n+1\right)=\Gamma \left(N_{D}\right)$.
