# Supplementary material for: HfZrO-based synaptic resistor circuit for a Super-Turing intelligent system
Source: Sci Adv. 2025 Feb 28;11(9):eadr2082. doi: 10.1126/sciadv.adr2082 (PMC13109932; doi:10.1126/sciadv.adr2082)
Supplement: Supplementary file 1 — Figs. S1 to S12 Legend for movie S1 [file sciadv.adr2082_sm.pdf]

Science Advances
AAAS

Supplementary Materials for

HfZrO-based synaptic resistor circuit for a Super-Turing intelligent system

Jungmin Lee *et al.*

Corresponding author: Yong Chen, yongchen@seas.ucla.edu

Sci. Adv. **11**, eadr2082 (2025)
DOI: 10.1126/sciadv.adr2082

The PDF file includes:

Figs. S1 to S12
Legend for movie S1

Other Supplementary Material for this manuscript includes the following:

Movie S1

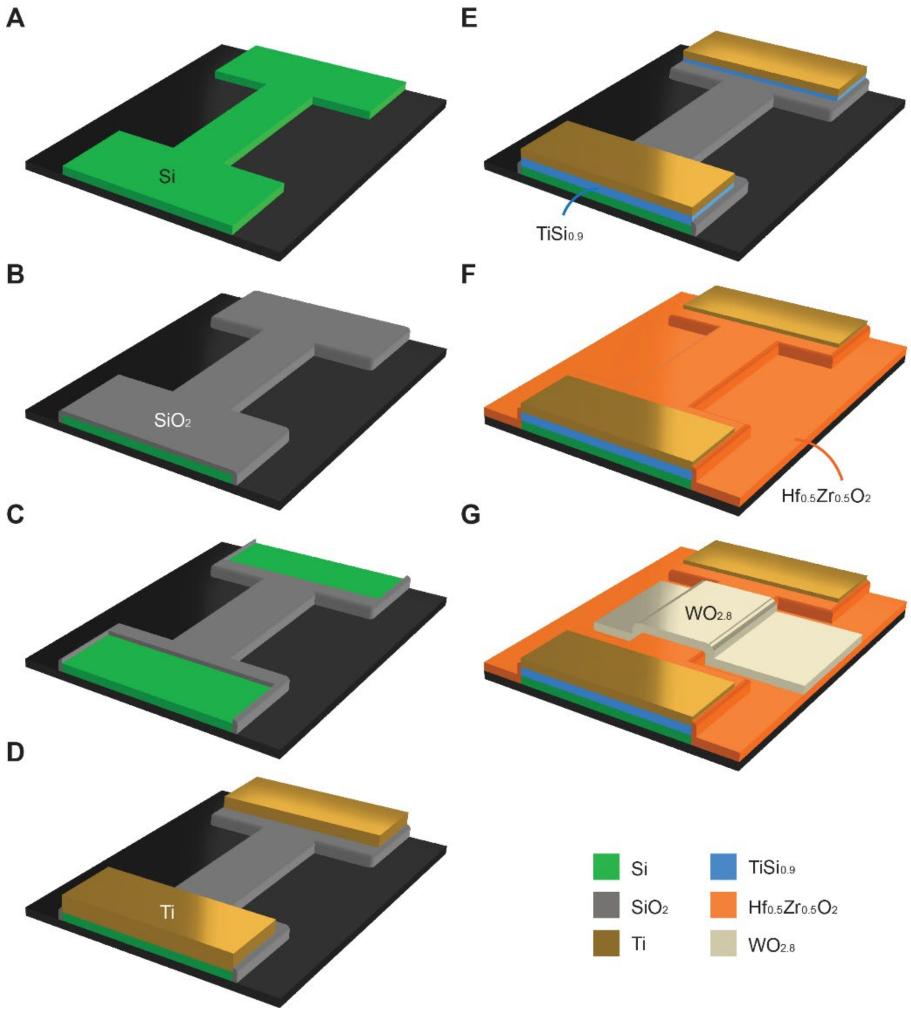

Fig. S1. The synstor circuit fabrication process. (**A**) A Si channel is etched on a SiO_2 surface. (**B**) The Si surface is oxidized and covered by a SiO_2 layer. (**C**) The SiO_2 layer in contact areas is etched. (**D**) Ti input and output electrodes are deposited in the contact areas, and (**E**) are annealed to form a titanium silicide ($TiSi_{0.9}$) layer sandwiched between the Si channel and Ti input/output electrodes. (**F**) A $Hf_{0.5}Zr_{0.5}O_2$ layer is deposited on the SiO_2 layer on the Si channel. **g**, A $WO_{2.8}$ reference electrode is made on the $Hf_{0.5}Zr_{0.5}O_2$ layer.

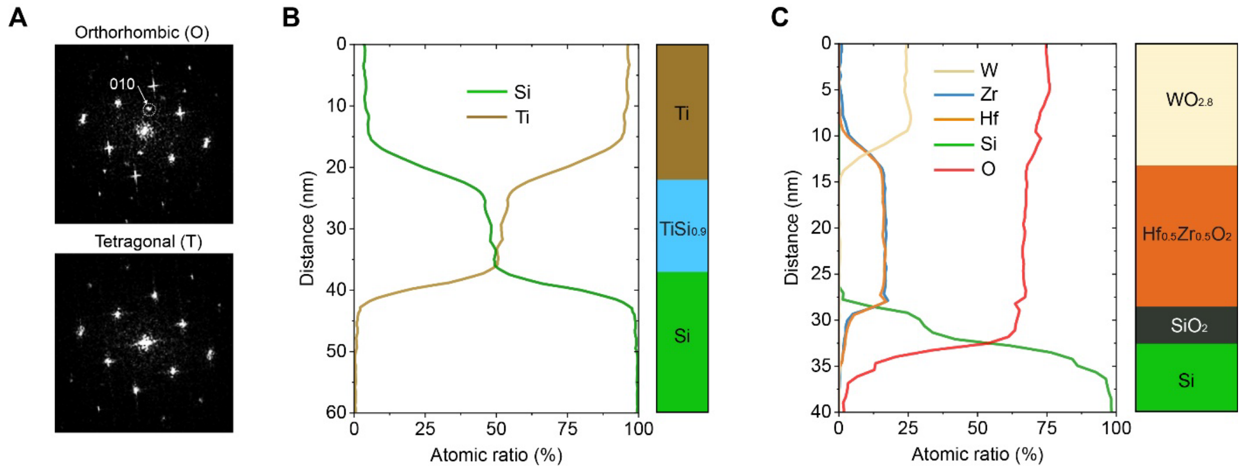

Fig. S2. Fast Fourier Transformation (FFT) patterns and composition profiles. (**A**) The FFT patterns from the STEM images of the orthorhombic (O) and tetragonal (T) phases in the $Hf_{0.5}Zr_{0.5}O_2$ layer shown in Fig. 1E. (**B**) The EDX composition profiles of the Ti/$TiSi_{0.9}$/Si heterojunction, (**C**) The EELS composition profiles of the $WO_{2.8}$/$Hf_{0.5}Zr_{0.5}O_2$/SiO_2/Si heterojunction.

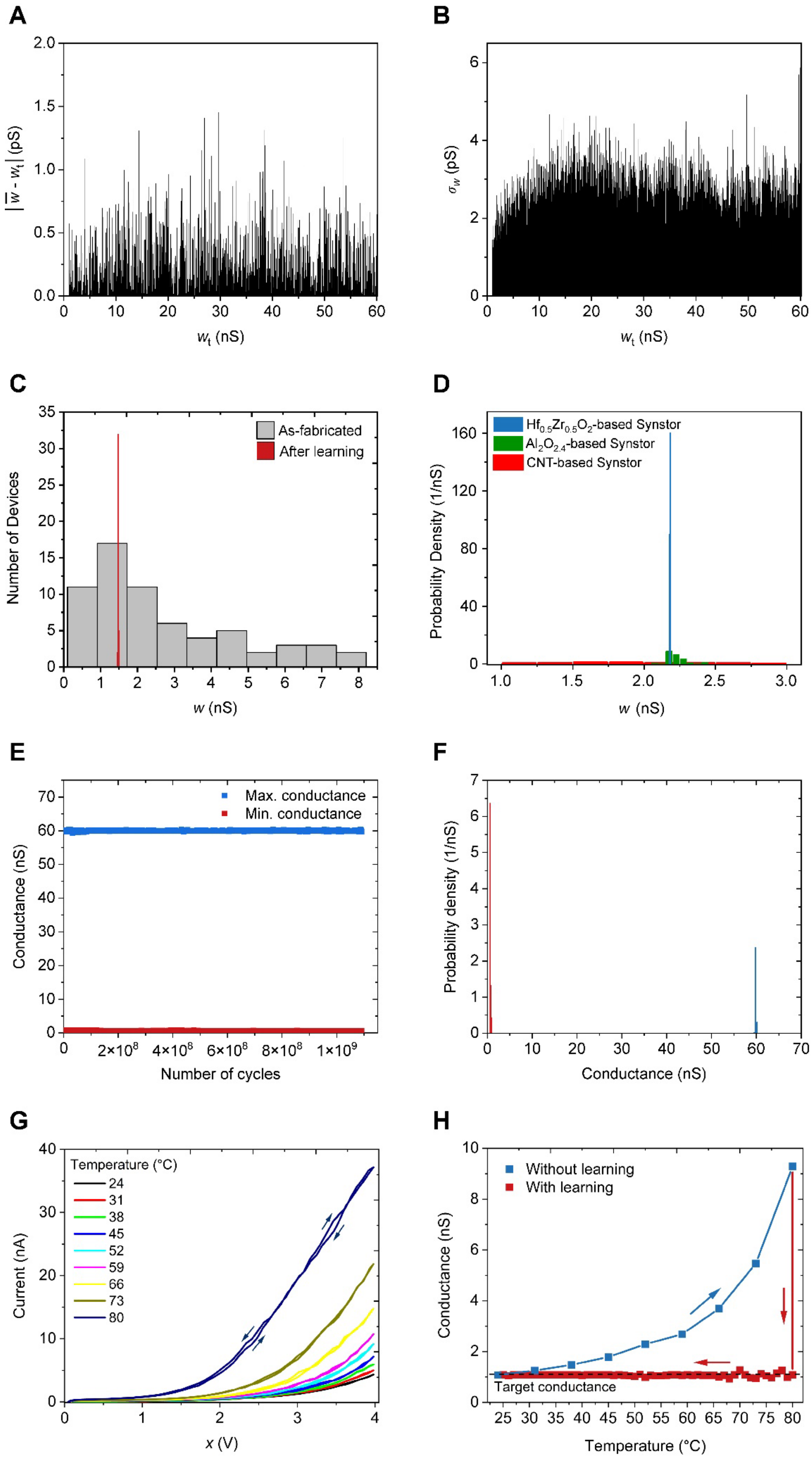

A
$|\bar{w} - w_t|$ (pS)
w_t (nS)
B
σ_w (pS)
w_t (nS)
C
As-fabricated
After learning
Number of Devices
w (nS)
D
$Hf_{0.5}Zr_{0.5}O_2$-based Synstor
$Al_2O_{2.4}$-based Synstor
CNT-based Synstor
Probability Density (1/nS)
w (nS)
E
Max. conductance
Min. conductance
Conductance (nS)
Number of cycles
F
Probability density (1/nS)
Conductance (nS)
G
Temperature (°C)
24
31
38
45
52
59
66
73
80
Current (nA)
x (V)
H
Without learning
With learning
Target conductance
Conductance (nS)
Temperature (°C)

Fig. S3. Learning accuracy, rate, endurance, and nonvolatile memory tests of synstors. Learning accuracy: (**A**) The absolute values of the differences between the average analog conductance values $\bar{w}$ and the targeted analog conductance values of synstors w_t, $|\bar{w} - w_t|$, and (**B**) the standard deviations of w, σ_w, are plotted against their corresponding targeted analog conductance values of synstors, w_t. (**C**) The number of devices is displayed versus synstor conductance values w for the 64 synstors, shown both as-fabricated (in red, with $\bar{w} = 2.695$ nS and $\sigma_w = 2.107$ nS) and after learning (in blue, with $\bar{w} = 1.531$ nS and $\sigma_w = 0.001$ nS) to a targeted conductance value ($w_t = 1.5315$ nS). (**D**) The probability densities of conductance values w of the $Hf_{0.5}Zr_{0.5}O_2$-based synstors (blue, this work), $Al_2O_{2.4}$-based synstors ($\bar{w} = 2.219$ nS and $\sigma_w = 0.062$ nS) (*37*) (green), and CNT- based synstors ($\bar{w} = 1.896$ nS and $\sigma_w = 0.450$ nS) (*35*) (red) are plotted after the synstors were tuned toward their targeted conductance values. (**E**) Synstor conductance values are plotted against modification cycles when the synstor was iteratively modified to reach its target maximum ($w_t = 60\, nS$, blue squares) and minimum ($w_t = 0.6\, nS$, red squares) conductance values. (**F**) The probability densities of the synstor conductance values are shown for the iterative modification to the target maximum (blue) and minimum (red) conductance values. (**G**) The current (I) through a synstor is plotted as a function of input voltage (x), where x is swept from 0 to 4 V and then back to 0, across temperatures ranging from 24 °C to 80 °C. (**H**) The synstor conductance (w) at $x = 4$ V is plotted against temperature, comparing the conductance without learning (blue) to that with learning (red) aimed at a target conductance value ($w_t = 1.08\, nS$).

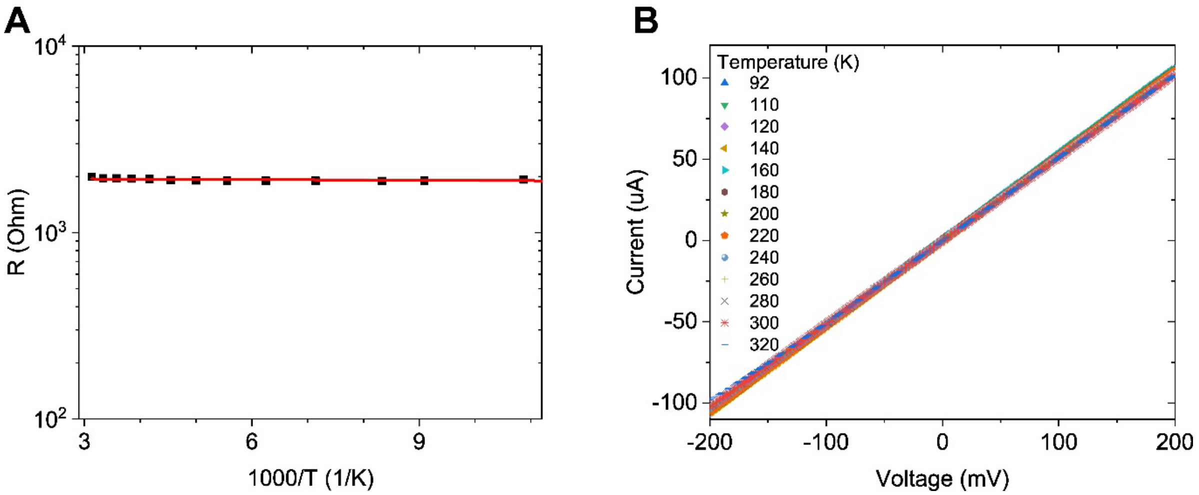

Fig. S4. The electric properties of $WO_{2.8}$ films. (**A**) Arrhenius plot of the resistance for a $WO_{2.8}$ film resistor, with the resistance shown on a logarithmic scale as a function of 1000/T. The experimental data (black squares) is best-fitted by a linear line (red). (**B**) The current through the $WO_{2.8}$ resistor is plotted against the applied voltage across temperatures ranging from $92\ K$ to $320\ K$.

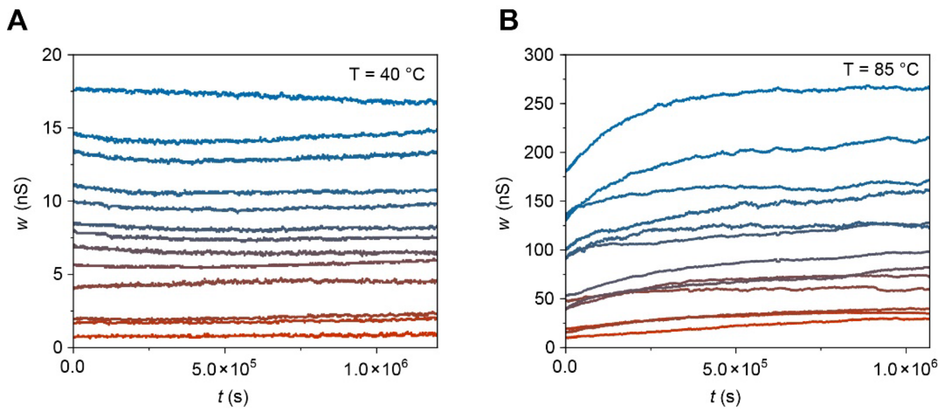

Fig. S5. Nonvolatile memory tests of synstors and device conductance comparison. After synstors were modified to different initial analog conductance values, the conductance value of each synstor, $w(t)$, measured at (**A**) $40\ °C$ and (**B**) $85\ °C$ is shown by dots in different colors as a function of time t over $10^6\ s$.

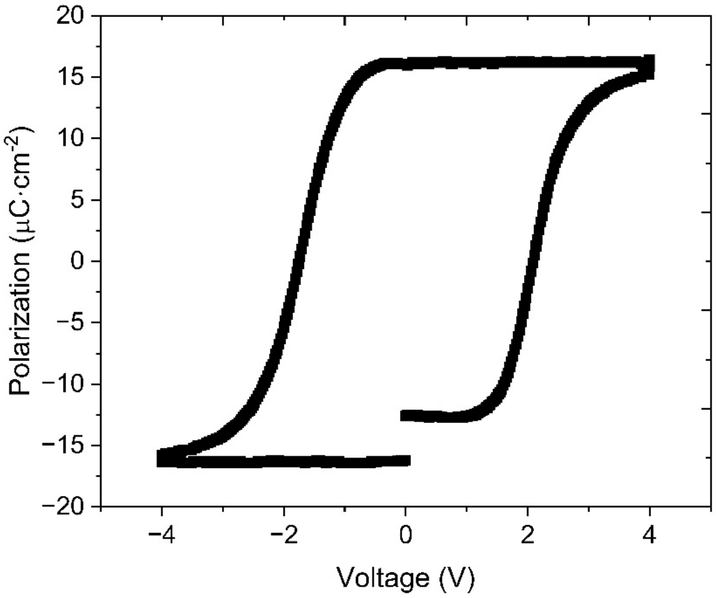

Fig. S6. Polarization-voltage (P-V) plots of a $Hf_{0.5}Zr_{0.5}O_2$ film. The polarization (P) of a 10 nm-thick HfZrO film $Hf_{0.5}Zr_{0.5}O_2$ film is shown as a function of the applied voltage across the film.

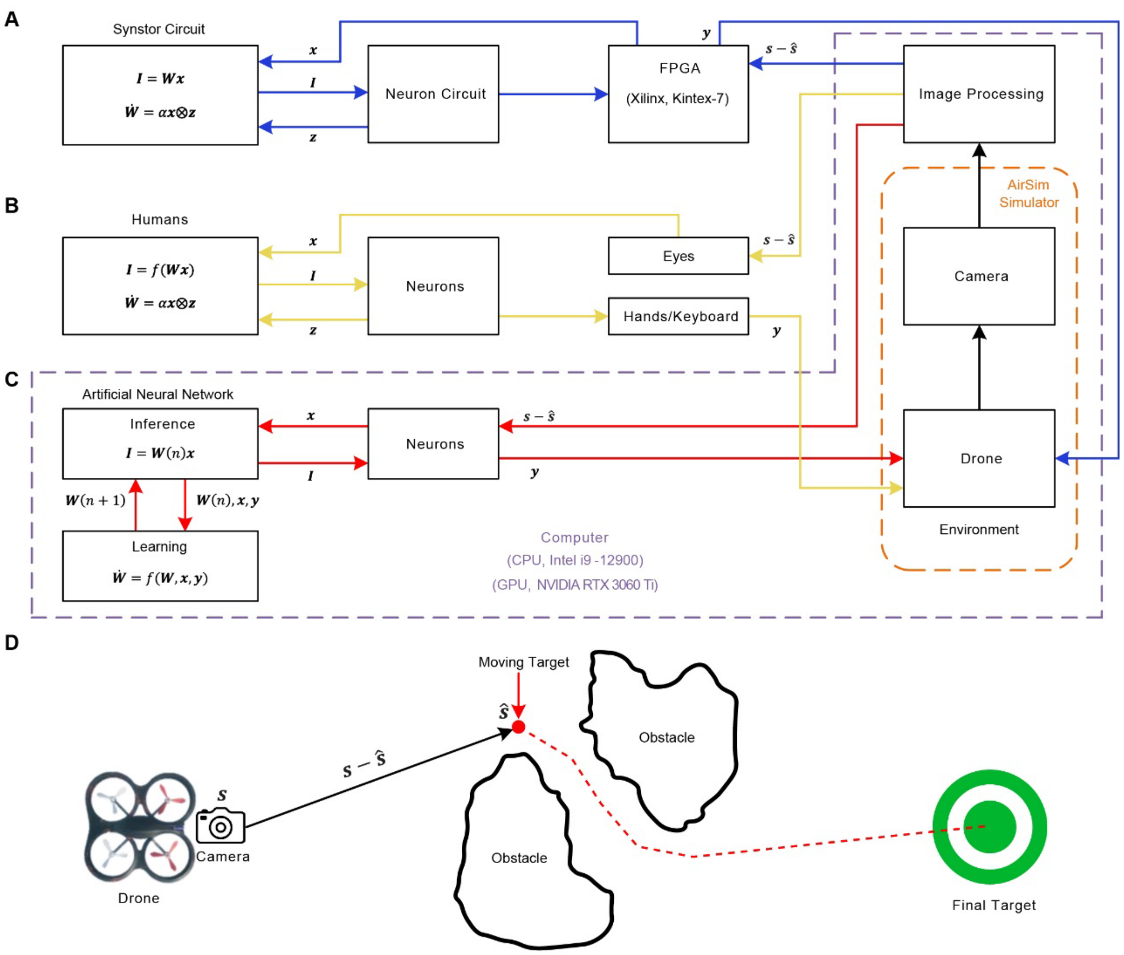

Fig. S7. Schematics of experimental systems. Experimental systems for (**A**) a synstor circuit with real-time learning, (**B**) human operators with real-time learning, and (**C**) a computer-based ANN with off-line learning to drive a drone toward a target position in a simulated environment. (**D**) Obstacles are detected by a simulated camera on the drone, a moving target position ($\hat{\boldsymbol{s}}$) is identified to avoid the obstacles, and the deviation of the drone position ($\boldsymbol{s}$) from its target position, $\boldsymbol{s} - \hat{\boldsymbol{s}}$, is input to the synstor circuit, operators, or ANN via interface circuits.

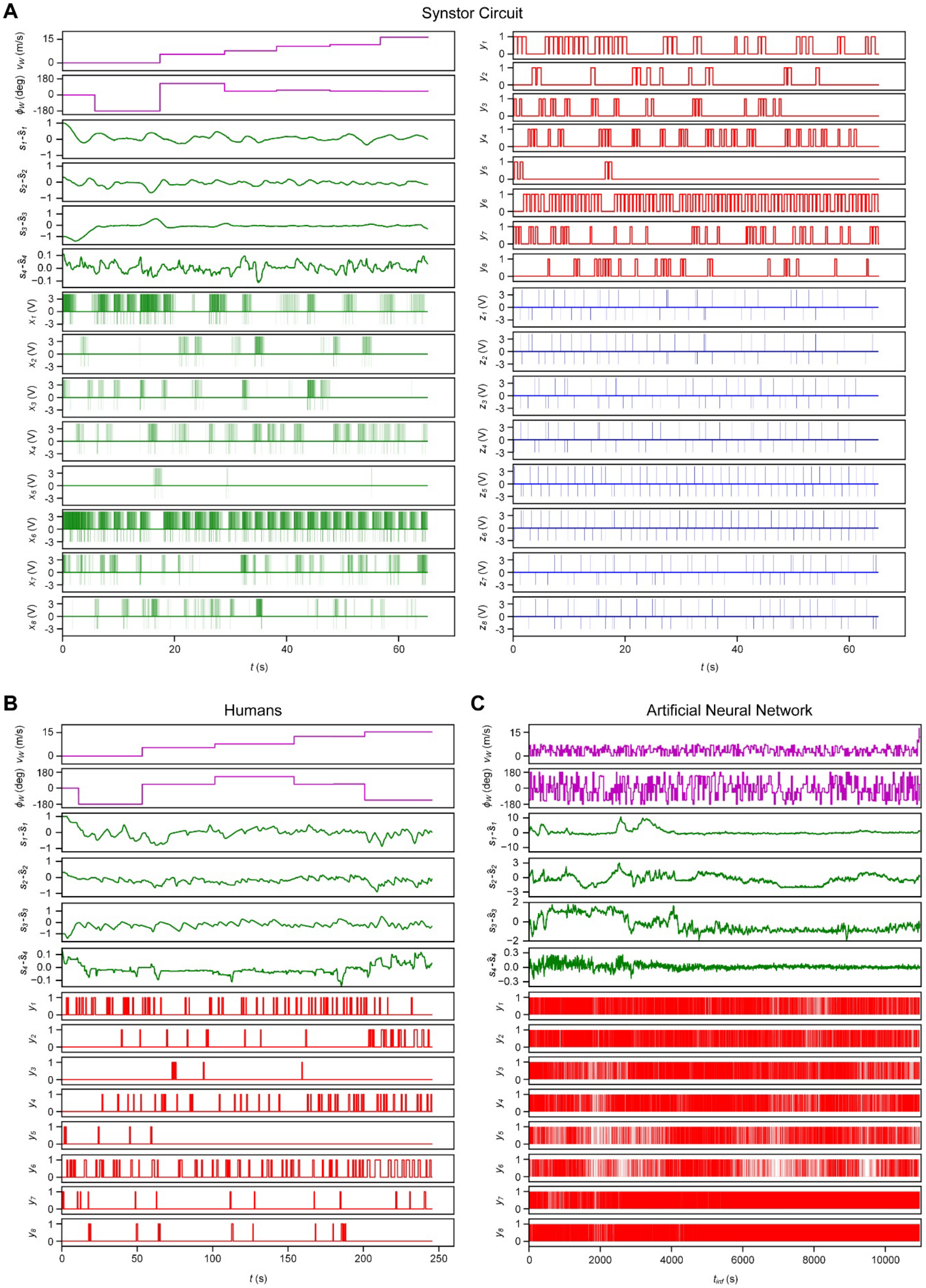
A
Synstor Circuit
B
Humans
C
Artificial Neural Network
v_W (m/s)
ϕ_W (deg)
s_1-$\hat{s}_1$
s_2-$\hat{s}_2$
s_3-$\hat{s}_3$
s_4-$\hat{s}_4$
x_1 (V)
x_2 (V)
x_3 (V)
x_4 (V)
x_5 (V)
x_6 (V)
x_7 (V)
x_8 (V)
y_1
y_2
y_3
y_4
y_5
y_6
y_7
y_8
z_1 (V)
z_2 (V)
z_3 (V)
z_4 (V)
z_5 (V)
z_6 (V)
z_7 (V)
z_8 (V)
t (s)
t_{inf} (s)

Fig. S8. Data from the experiments with a drone driven by a synstor circuit, a human operator, and an ANN running on a computer. The wind speed V_W, the wind direction measured in degrees clockwise from north ϕ_W, the deviations of the drone position from the local target measured along the left-right $s_1 - \hat{s}_1$, up-down $s_2 - \hat{s}_2$, forward-backward $s_3 - \hat{s}_3$, clockwise-counterclockwise yaw $s_4 - \hat{s}_4$, directions, and the actuation pulses, y_1, y_2, y_3, y_4, y_5, y_6, y_7, and y_8 to move the drone along left, right, up, down, forward, backward, clockwise, and counterclockwise yaw directions are displayed against the time t in the experiments with a drone driven by (**A**) a synstor circuit, (**B**) a human operator, and (**C**) an ANN running on a computer. The input voltage pulses x_1, x_2, x_3, x_4, x_5, x_6, x_7, and x_8 converted from the $\boldsymbol{s} - \hat{\boldsymbol{s}}$ signals along left, right, up, down, forward, backward, clockwise, and counterclockwise yaw directions, respectively, and the back-propagating voltage pulses z_1, z_2, z_3, z_4, z_5, z_6, z_7, and z_8 on the output electrodes associated with the actuation pulses, y_1, y_2, y_3, y_4, y_5, y_6, y_7, and y_8, respectively, are also displayed against the time t.

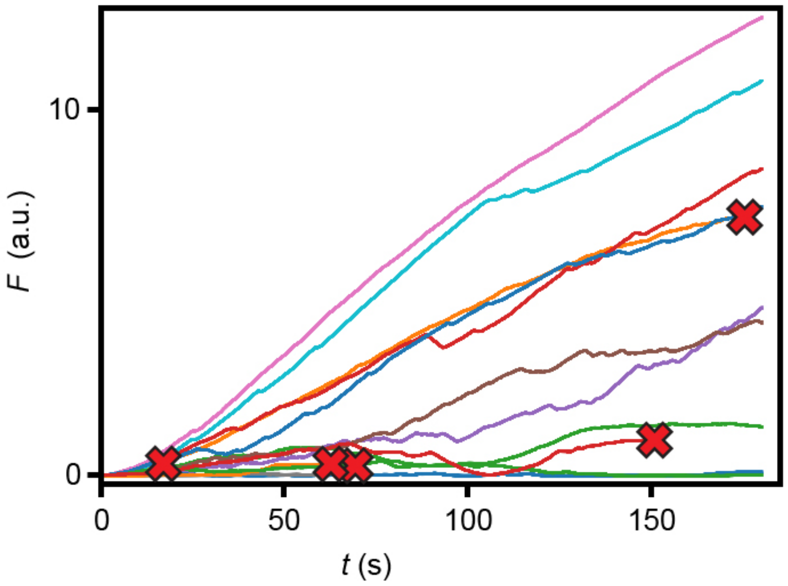

Fig. S9. Experiments to drive a drone by a synstor circuit without learning. During the flight processes of a drone driven by a synstor circuit with its conductance matrix set to random values and no learning, the objective functions, $F = \frac{1}{2}(\boldsymbol{s} - \hat{\boldsymbol{s}})^2$, with $\boldsymbol{s} - \hat{\boldsymbol{s}}$ as the deviations of the drone position ($\boldsymbol{s}$) from its target position ($\hat{\boldsymbol{s}}$) are displayed versus flight time t. The red crash symbols indicate that the drone crashed into the tree.

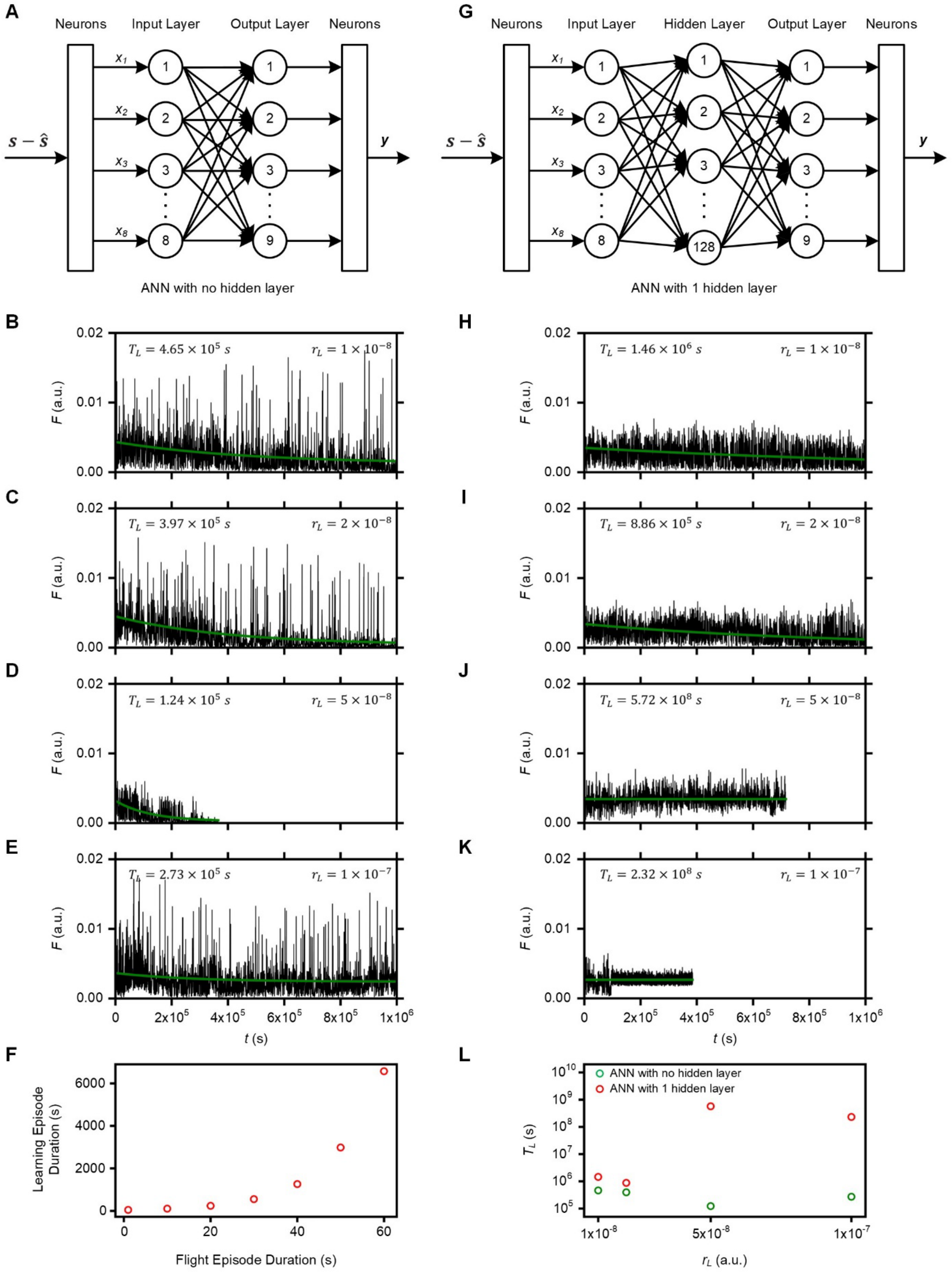
A
Neurons
Input Layer
Output Layer
Neurons
s − ŝ
y
ANN with no hidden layer
G
Neurons
Input Layer
Hidden Layer
Output Layer
Neurons
s − ŝ
y
ANN with 1 hidden layer
B
$T_L = 4.65 \times 10^5\ s$
$r_L = 1 \times 10^{-8}$
C
$T_L = 3.97 \times 10^5\ s$
$r_L = 2 \times 10^{-8}$
D
$T_L = 1.24 \times 10^5\ s$
$r_L = 5 \times 10^{-8}$
E
$T_L = 2.73 \times 10^5\ s$
$r_L = 1 \times 10^{-7}$
H
$T_L = 1.46 \times 10^6\ s$
$r_L = 1 \times 10^{-8}$
I
$T_L = 8.86 \times 10^5\ s$
$r_L = 2 \times 10^{-8}$
J
$T_L = 5.72 \times 10^8\ s$
$r_L = 5 \times 10^{-8}$
K
$T_L = 2.32 \times 10^8\ s$
$r_L = 1 \times 10^{-7}$
F (a.u.)
t (s)
F
Learning Episode Duration (s)
Flight Episode Duration (s)
L
ANN with no hidden layer
ANN with 1 hidden layer
T_L (s)
r_L (a.u.)

Fig. S10. Learning experiments to drive a drone by ANN with different structures and learning parameters. (**A**) a schematic shows the structure of an ANN with 8 neurons at its input layer, no hidden layer, and 9 neurons at its output layer. The local objective functions, $F_L = \frac{1}{2}\boldsymbol{s}_L^2$, with $\boldsymbol{s}_L$ as the deviations of the drone position from its local target position, are displayed versus accumulative flight and learning time t during the flight processes of the drone driven by the ANN shown in (**A**) with a learning rate of r_L equal to (**B**) 10^{-8}, (**C**) 2×10^{-8}, (**D**) 5×10^{-8}, and (**E**) 10^{-7}. (**F**) the duration of learning episode to execute learning function based on the data from a flight episode is shown against the duration of flight episode. (**G**) A schematic shows the structure of an ANN with 8 neurons at its input layer, 128 neurons at its hidden layer, and 9 neurons at its output layer. The objective functions, $F = \frac{1}{2}(\boldsymbol{s} - \hat{\boldsymbol{s}})^2$, are displayed versus accumulative flight and learning time t during the flight processes of the drone driven by the ANN shown in (**G**) with a learning rate of r_L equal to (**H**) 10^{-8}, (**I**) 2×10^{-8}, (**J**) 5×10^{-8}, and (**K**) 10^{-7}. The $F - t$ curves are best-fitted by $F(t) = (F(0) - F_e)\, e^{-t/T_L} + F_e$ (green lines) to extrapolate the average learning times T_L. (**L**) T_L is displayed versus the leaning rate r_L in the ANN shown in (**A**) (with no hidden layer, green circles) and (**G**) (with 1 hidden layer, red circles).

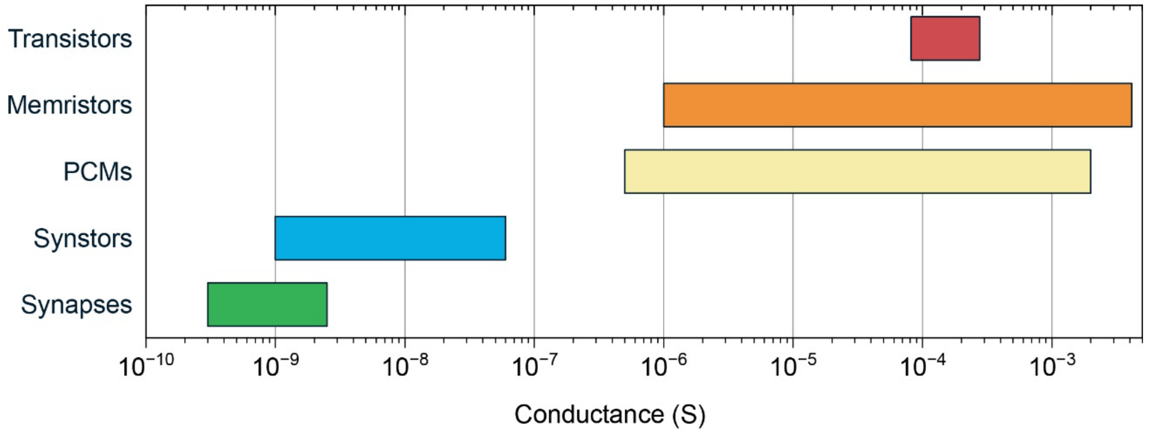

Fig. S11. Conductance comparison. The conductance of synstors in this work is compared with that of synapses (*28*), transistors (*5-7, 48*), memristors (*15, 17, 49, 50*), and phase change memory resistors (*22*).

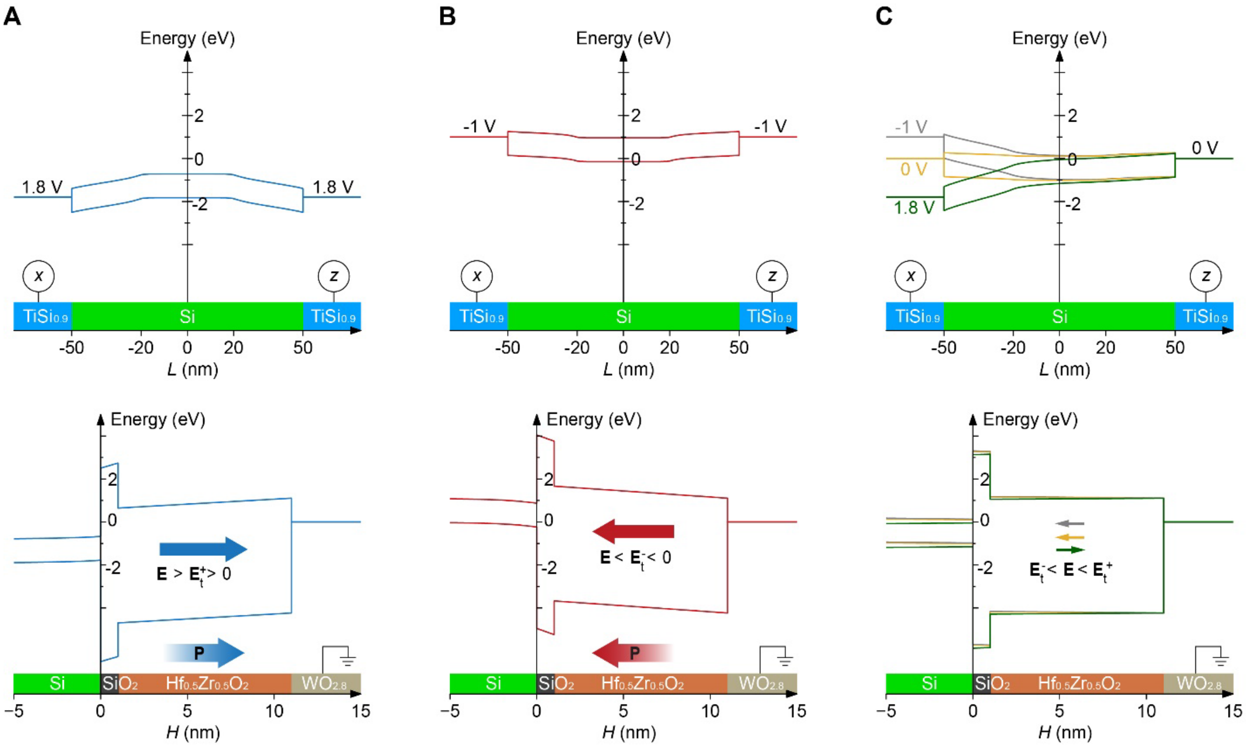

Fig. S12. The energy-band structures of a synstor with a channel length of 100 nm. The simulated energy-band structures of the $TiSi_{0.9}$ input electrode, Si channel, and $TiSi_{0.9}$ output electrode along the L axis in fig. S7A, and of the grounded $WO_{2.8}$ reference electrode, a 10 nm-thick $Hf_{0.5}Zr_{0.5}O_2$ layer, an 1 nm-thick SiO_2 dielectric layer, and Si channel along the H axis in fig. S7A of a synstor with a channel length of 100 nm under the conditions of (**A**) the potential at the input electrode $x = 1.8\,V$, the potential at the output electrode $z = 1.8\,V$, (**B**) $x = z = -1.0\,V$, and (**C**) $x = z = 0\,V$ (yellow lines), $x = -1.0\,V$ and $z = 0$ (deep blue lines), and $x = 1.8\,V$ and $z = 0$ (green lines). When the electric field, $\boldsymbol{E}$, in the $Hf_{0.5}Zr_{0.5}O_2$ layer satisfies $\boldsymbol{E} > \boldsymbol{E}_t^+ > 0$ in **a**, or $\boldsymbol{E} < \boldsymbol{E}_t^- < 0$ in **b**, with $\boldsymbol{E}_t^+$ and $\boldsymbol{E}_t^-$ as the positive and negative electric threshold fields to switch the dipole, $\boldsymbol{P}$, of the ferroelectric domains in the $Hf_{0.5}Zr_{0.5}O_2$ layer, $\boldsymbol{P}$ is modified to align along $\boldsymbol{E}$. When $\boldsymbol{E}_t^- < \boldsymbol{E} < \boldsymbol{E}_t^+$ in (**C**), thus $\Delta\boldsymbol{P} \approx 0$.

Movie S1. Experiments to negative a drone by a synstor circuit, human operator, and ANN. A drone driven by (**A**), a synstor circuit, (**B**) a human operator with real-time learning, and (**C**) an ANN running in a computer with off-line learning toward a target (an enemy drone) by avoiding obstacles in an environment with aerodynamically changing wind. (Top) Videos show that the drone flying toward local targeted positions marked by red arrows. (Middle) The flying traces of the drone in the environment are displayed. (Bottom) Objective functions, $F = \frac{1}{2}(\boldsymbol{s} - \hat{\boldsymbol{s}})^2$, with $\boldsymbol{s} - \hat{\boldsymbol{s}}$ as the deviations of the drone position ($\boldsymbol{s}$) from its target position ($\hat{\boldsymbol{s}}$) versus time t. The changing wind speeds and directions in the environment are indicated. The target position is marked by a green target symbol. The red crash symbol indicates that the drone crashed into the tree.
